# PWWP domain-containing protein Crf4-3 specifically modulates fungal azole susceptibility by regulating sterol C-14 demethylase ERG11

**DOI:** 10.1128/msphere.00703-24

**Published:** 2024-12-13

**Authors:** Pengju Yu, Shuting Ye, Mi Zhou, Long Zhang, Zhongchi Zhang, Xianyun Sun, Shaojie Li, Chengcheng Hu

**Affiliations:** 1 Institute of Microbiology, Chinese Academy of Sciences 85387 , Beijing, China; 2 College of Life Sciences, University of Chinese Academy of Sciences, Beijing, China; 3 Shandong Jinniu Group Co., Ltd., Jinan, China; 4 Shanghai Fondin Bio_Tech Co., Ltd., Shanghai, China; University of Georgia, Athens, Georgia, USA

**Keywords:** PWWP domain, azole resistance, transcriptional regulation, ergosterol biosynthesis, *Neurospora crassa*, *Aspergillus fumigatus*

## Abstract

The widespread use of azole antifungals in agriculture and clinical settings has led to serious drug resistance. Overexpression of the azole drug target 14α-demethylase ERG11 (CYP51) is the most common fungal resistance mechanism. However, the presence of additional regulatory proteins in the transcriptional response of *erg11* is not yet fully elucidated. In this study, leveraging the identified key promoter region of *erg11* that controls its response to azoles in *Neurospora crassa*, we pinpointed a protein, Crf4-3, which harbors a PWWP domain and exerts a positive regulatory influence on azole resistance, as determined by DNA pulldown assays. The removal of Crf4-3 results in heightened sensitivity to azoles while remaining unaffected by other stressors tested. Additionally, the deletion leads to the abolition of transcriptional responses of genes such as *erg11* and *erg6* to ketoconazole. Interestingly, the basal expression of *erg1*, *erg11*, *erg25*, and *erg3A* is also affected by the deletion of *crf4-3*, indicating its role in sterol homeostasis. Crf4-3 homologs are broadly distributed across the *Pezizomycotina* fungi. The gene deletion for its homologous protein in *Aspergillus fumigatus* also significantly improves sensitivity to azoles such as voriconazole, primarily through the attenuation of the transcriptional response of *erg11*. Our data, for the first time, identified Crf4-3 as a novel regulatory protein in the azole stress response of filamentous fungi, offering fresh insights into the mechanisms of azole resistance.

**IMPORTANCE:**

Transcriptional control of pivotal genes, such as *erg11*, stands as the primary driver of azole resistance. Although considerable effort has been dedicated to identifying transcription factors involved, our knowledge regarding the use of transcriptional regulation strategies to combat azole resistance is currently limited. In this study, we reveal that a PWWP domain-containing protein Crf4-3, which is conserved in *Pezizomycotina* fungi, modulates fungal azole sensitivity by transcriptionally regulating sterol biosynthetic genes, including *erg11*. These results also broaden the understanding of fungal PWWP domain-containing proteins regarding their roles in regulating resistance against azole antifungals. Considering research on small molecules targeting the PWWP domain in humans, Crf4-3 homolog emerges as a promising target for designing fungal-specific drugs to combat azole resistance.

## INTRODUCTION

Fungal pathogens pose a severe threat to human health and food security (1–3). It is estimated that nearly 300 million people worldwide suffer from fungal diseases, with more than 1.5 million deaths annually (4, 5). Specific pathogenic fungi are also implicated in the development of cancer (6–8). In agriculture, fungi account for over 70% of the pathogens causing crop diseases, and the damage they cause annually could feed 600 million people at the basic food level (9, 10). Azoles are the most commonly employed class of antifungal agents in a limited arsenal. They target sterol 14α-demethylase, an enzyme encoded by *erg11*, thereby disrupting the synthesis of ergosterol, a vital component of the fungal cell membrane, as a means to combat fungal infections (11–13). However, in response to azoles, fungi can adapt to drug-induced stress by globally modulating their transcriptome, resulting in widespread changes in gene expression across the entire genome (14–16). Among them, the upregulation of *erg11* and efflux pump-related genes stands out as a pivotal factor contributing to azole resistance, as evidenced by research in laboratory settings and clinical resistant isolates (17, 18). These findings underscore the critical role of transcriptional control over key genes in the development of azole resistance. Therefore, understanding the regulatory mechanisms of fungal transcriptional responses to azoles holds theoretical implications for addressing drug resistance issues.

The majority of research investigating the transcriptional response mechanisms to azoles has centered on the role of transcription factors (19–21). For instance, in *Candida* species, azole treatment activates the transcription factor Upc2p, which in turn triggers transcriptional responses, including the upregulation of *erg11*. Gain-of-function mutations in Upc2p are frequently implicated in the overexpression of *erg11*, which is a key driver of azole resistance observed in clinical isolates (22). In *Schizosaccharomyces pombe*, the transcription factor involved in regulating *erg11* expression is the SREBP protein Sre1 (23). However, in *Fusarium graminearum*, a new transcription factor, FgSR, plays a crucial role in *erg11* transcriptional response (24). In *Neurospora crassa* and *Aspergillus fumigatus*, the regulatory mechanism governing *erg11* expression is more intricate, involving a network of factors including ADS-1, CCG-8, and CSP-1 in *N. crassa* and SrbA, AtrR, SltA, and the CBC (CCAAT-binding complex) in *A. fumigatus* (25–29). Transcription factors that govern the expression of genes encoding drug efflux pumps have also been identified and demonstrate species-specific regulatory patterns across different fungal taxa (25, 30, 31). Due to the efforts of these fungi, our understanding of azole-induced transcriptional regulation has been improved.

Transcriptional regulation of gene expression is a sophisticated and dynamic process involving a complex series of biophysical events. This necessitates the precise engagement of DNA-protein interactions, the recruitment, and organization of transcriptional machinery complexes, along with the restructuring of chromatin architecture and the imposition of epigenetic modifications. Consequently, a vast array of molecules is essential, including transcription factors, cofactors, and chromatin-modulating factors, to facilitate this intricate regulatory process. However, in the context of the azole stress response, the intricacies of transcriptional regulation remain only partially understood, especially in filamentous fungi. Beyond the involvement of transcription factors, previous studies have only highlighted the pivotal role of histone modifications in regulating resistance to azoles (32–34). The presence of additional regulatory proteins in this process is unclear. Furthermore, transcription factors have traditionally been regarded as “undruggable” targets (35, 36). Significant exertions are required to harness transcription factors as a means to combat antifungal resistance (37–39). Therefore, it is intriguing to explore whether there exist additional and more conserved and druggable transcriptional regulators that play a role in responses to azole stress.

In this study, using *N. crassa* as a model, our initial objective was to identify transcription factors that directly regulate *erg11* expression through DNA pulldown. Unexpectedly, we uncovered a novel regulator, Crf4-3, that is implicated in the modulation of azole stress response. Crf4-3, a protein with a Pro-Trp-Trp-Pro (PWWP) domain, functions in nuclear to positively regulate the transcriptional responses of sterol synthesis genes, such as *erg11* and *erg6*, to azole stress, thereby specifically influencing the fungal susceptibility to azoles. Protein sequence and phylogenetic analysis indicate that Crf4-3 is highly conserved in *Pezizomycotina* fungi. In *A. fumigatus*, the homologous protein of Crf4-3 similarly modulates the transcriptional response of *erg11* and impacts sensitivity to azoles. With extensive research on small molecules targeting the PWWP domain (40), Crf4-3 homolog is a promising fungal-specific target for drug design aimed at combating azole resistance challenges.

## RESULTS

### The −431 to −420 region of the *erg11* promoter in *N. crassa* is critical for its response to azole drugs

**Fig 1:**
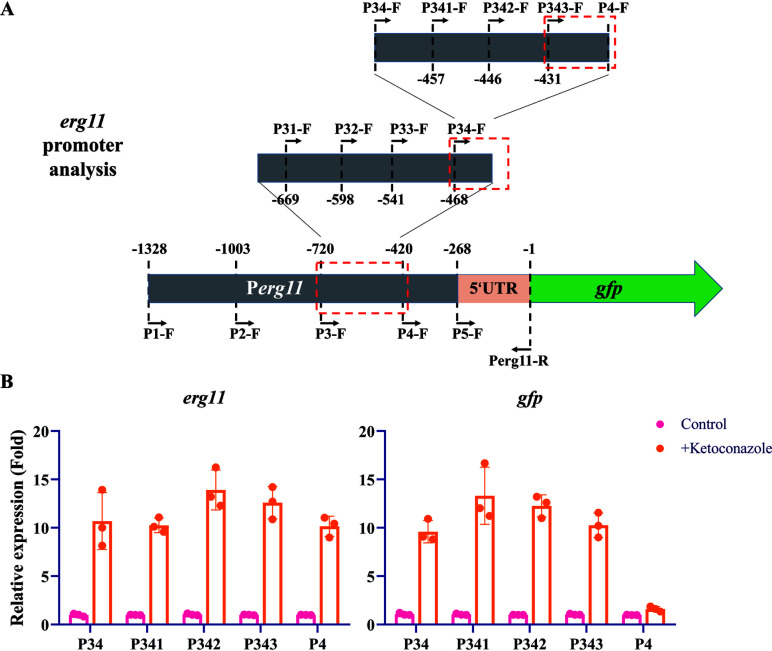
The −431 to −420 region of the *erg11* promoter in *N. crassa* is critical for its response to azole drugs. (**A**) Illustrative schematic depicting the sequential deletion process of the *erg11* promoter region using the *gfp* gene as a reporter. (**B**) Gene expression detection of *erg11* and *gfp* in strains P34 (*gfp* driven by the −468 to 0 region of the *erg11* promoter), P341 (*gfp* driven by the −457 to 0 region of the *erg11* promoter), P342 (*gfp* driven by the −446 to 0 region of the *erg11* promoter), P343 (*gfp* driven by the −431 to 0 region of the *erg11* promoter), and P4 (*gfp* driven by the −420 to 0 region of the *erg11* promoter) after treating with 1.5 µg/mL ketoconazole for 12 h. Gene expression was detected using qRT-PCR. Expression levels were calculated using the 2^–ΔΔCT^ method with the expression of the *β-tubulin* gene as an internal reference. The expression level of each gene was normalized to one under untreated conditions. Error bars indicate standard deviations, *n* = 3.

In previous studies using *N. crassa* as a model, we identified multiple transcription factors in fungi that participate in the stress response to antifungal azoles (25–27). However, these transcription factors have limited regulatory effects on the expression of genes such as *erg11* in *N. crassa*. It is possible that these transcription factors function coordinately or there exist other key regulatory proteins involved. To address these presumptions, we attempted to identify proteins that might directly regulate the expression of azole-responsive genes. Azole target encoding gene *erg11* was chosen because of its importance in azole resistance. We adopted a reverse genetic strategy by first determining the key promoter region of *erg11* in *N. crassa* that responds to azole drugs. It is proposed that upon deletion of the key promoter region, *erg11* should lose its ability to respond to azoles. Based on this rationale, we constructed a *gfp* reporter system, utilizing the *erg11* promoter to drive *gfp* expression (Fig. 1A), and integrated it into the *his-3* locus in the genome. At the same time, we monitored the expression of the *erg11* gene to ensure that the strain was in a state of normal response to azole treatment. When the full-length 1328 bp *erg11* promoter was used to drive *gfp* expression, *gfp* was able to produce a transcriptional response to ketoconazole treatment, with a magnitude comparable to that of *erg11* (Fig. S1). We then performed multiple stepwise deletions on the *erg11* promoter within the reporter system (Fig. 1A). By detecting the expression of *erg11* and *gfp* genes, we ultimately determined that the −431 to −420 region (-TCCGAATACGA-) of the *erg11* promoter is the key region for azole response. Under conditions where the *erg11* gene could produce about 10-fold transcriptional upregulation in response to ketoconazole, the *gfp* reporter gene completely lost its response to ketoconazole when this key promoter region was removed (Fig. 1B). Interestingly, upon FIMO (Find Individual Motif Occurrences) analysis, similar sites to this critical region were identified in the *erg11* promoter regions of ascomycetes, especially filamentous fungi (Data set S1). Among the identified sites, a subset was identified as potential binding sites for transcription factors such as FgSR (24) and Upc2p (41), which are essential for the regulation of sterol biosynthesis (Data set S1). These findings suggest that the response of the *erg11* gene in *N. crassa* to azoles requires the presence of the −431 to −420 region of the promoter, which may be a recognition site for key regulatory proteins.

### DNA pulldown identifies Crf4-3 as a novel protein regulating *erg11* response to azole drugs

**Fig 2:**
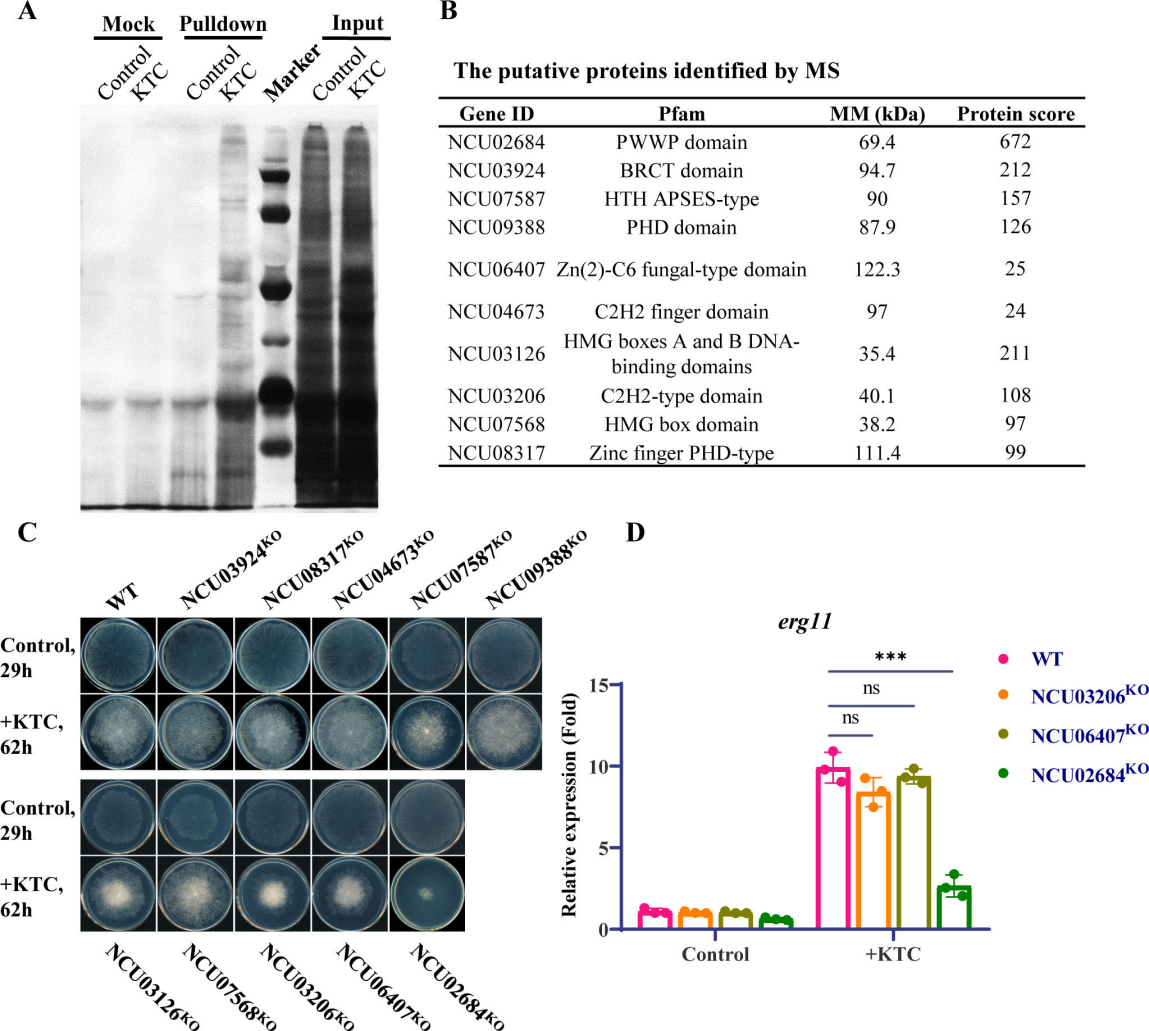
DNA pulldown identifies Crf4-3 as a novel protein regulating *erg11* response to azole drugs. (**A**) Biotin-based DNA pulldown assay. A fragment of biotinylated dsDNA containing the −431 to −420 of region *erg11* promoter was used as bait for pulldown assay. Nuclear protein extracted from ketoconazole-treated and non-treated mycelia were used. Input: the original nuclear protein extracts; Mock: eluted protein fractions from a mock DNA pulldown assay using the streptavidin magnetic beads coupled without biotinylated dsDNA; Pulldown: eluted protein fractions from DNA pulldown assay using streptavidin magnetic beads coupled with dsDNA; KTC, Ketoconazole. Protein bands presented in the pulldown assay, but not mock assy, were cut from the gel, trypsin-digested, and submitted to mass spectrometry. (**B**) Identified putative proteins with DNA binding domain. (**C**) Drug sensitivity test for the knockout mutations of identified transcription factors. A 2.5 µL conidial suspension (2 × 10^6^ spores/mL) of each strain was inoculated onto the center of Vogel’s plates with or without 1.5 µg/mL ketoconazole and incubated in the dark at 28°C. WT: wild-type strain. (**D**) Gene expression detection of *erg11* in NCU03206^KO^, NCU06407^KO^, and NCU02684^KO^ strains treated with or without 1.5 µg/mL ketoconazole for 12 h. Gene expression was detected using qRT-PCR. Expression levels were calculated using the 2^–ΔΔCT^ method with the expression of the *β-tubulin* gene as an internal reference. The expression level of *erg11* in WT without treatment was normalized to 1. The levels of significance for differences between the two samples are indicated by asterisks (*, *P* < 0.05; **, *P* < 0.01; ***, *P* < 0.001; ns, not significant, *P* > 0.05). Error bars indicate standard deviations, *n* = 3.

To obtain proteins that may participate in the regulation of *N. crassa erg11* response to azole drugs, DNA pulldown coupled with MS analysis using a DNA fragment containing the key region of the *erg11* promoter as a probe was conducted (Fig. 2A). By screening for proteins with DNA-binding domains, as shown in Fig. 2B, 10 potential regulatory factors were identified. Unexpectedly, transcription factors identified previously were not found in the list. Among them, the protein Crf4-3 (NCU02684) with a predicted PWWP domain had the highest score in the mass spectrometry results. High expression of *erg11* is one of the main reasons for fungal resistance to azole drugs. If these proteins are involved in the transcriptional response of *erg11* to azoles, their knockout would lead to hypersensitivity to azole drugs. Therefore, we examined the azole sensitivity on plates for knockout strains of these proteins. Ketoconazole was chosen as a standard azole for the screening process and downstream expression analysis, owing to its efficacy against *N. crassa* and its cost-effectiveness. The results showed that the knockout of *crf4-3* (NCU02684), NCU03206, and NCU06407 genes caused the strains to grow slower on plates containing ketoconazole, compared with WT (Fig. 2C; Fig. S2). In particular, the phenotype of the *crf4-3* knockout strain was the most pronounced, with a colony size of only 20% of the wild type, showing a hypersensitive phenotype. Further qRT-PCR analysis of *erg11* gene expression showed that although the expression level of wild-type *erg11* was upregulated by about 9-fold after treatment with ketoconazole, the transcriptional response of *erg11* after *crf4-3* knockout was only about 3-fold (Fig. 2D), indicating that Crf4-3 may be involved in the transcriptional response of *erg11* to azole stress. The knockout of NCU03206 and NCU06407 did not affect the transcriptional response of *erg11* to ketoconazole (Fig. 2D).

**Fig 3:**
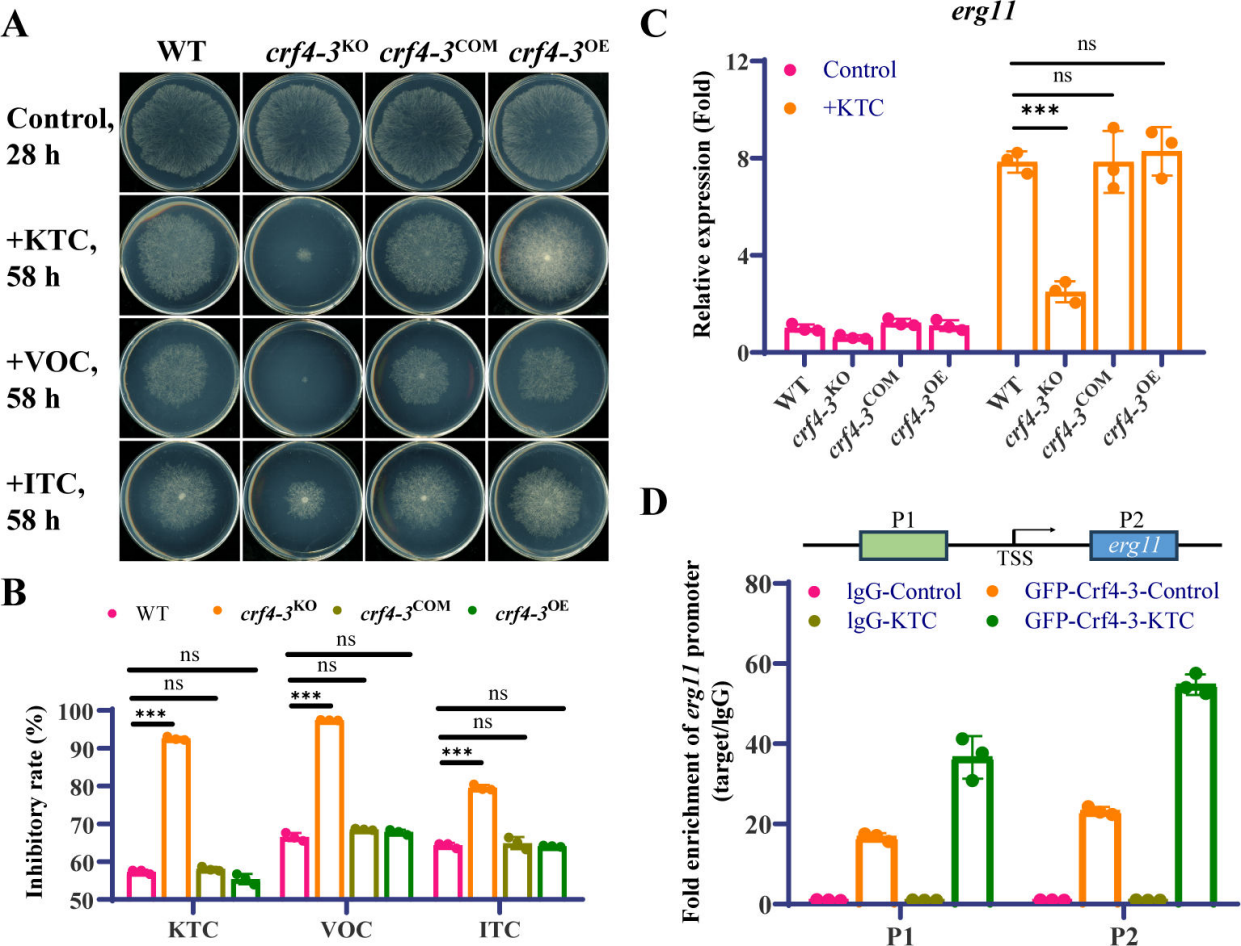
Crf4-3 knockout specifically affects sensitivity to azoles. (**A**) Drug sensitivity test of *crf4-3*^KO^, *crf4-3*^COM^, and *crf4-3*^OE^ strains to three different azole drugs. A 2.5 µL conidial suspension (2 × 10^6^ spores/mL) of each strain was inoculated onto the center of Vogel’s plates with or without azoles and incubated in the dark at 28°C. The concentrations of the azoles used were 1.5 µg/mL for ketoconazole (KTC), 0.5 µg/mL for voriconazole (VOC), and 20 µg/mL for itraconazole (ITC). (**B**) Rates of inhibition of the wild type, *crf4-3*^KO^, *crf4-3*^COM^, and *crf4-3*^OE^ strains by KTC, VOC, and ITC. The inhibition rate was calculated as 1 − [(colony diameter in drug plate/growth time)/(colony diameter in control plate/growth time)]. The levels of significance for differences between the two strains are indicated by asterisks (*, *P* < 0.05; **, *P* < 0.01; **, *P* < 0.001; ns, not significant, *P* > 0.05). Error bars indicate standard deviations, *n* = 3. (**C**) Gene expression detection of *erg11* in *crf4-3*^KO^, *crf4-3*^COM^, and *crf4-3*^OE^ strains treated with 1.5 µg/mL ketoconazole for 12 h. Gene expression was detected using qRT-PCR. Expression levels were calculated using the 2^–ΔΔCT^ method with the expression of the *β-tubulin* gene as an internal reference. The expression level of *erg11* in the wild type without treatment was normalized to 1. The levels of significance for differences between the two strains are indicated by asterisks (*, *P* < 0.05; **, *P* < 0.01; ***, *P* < 0.001; ns, not significant, *P* > 0.05). Error bars indicate standard deviations, *n* = 3. (**D**) The binding ability of Crf4-3 to the promoter of *erg11 in vivo* using ChIP-qPCR. The mycelium was treated with or without 1.5 µg/mL ketoconazole for 6 h. IP with rabbit lgG was used as a control. *n* = 3.

To further confirm the function of Crf4-3, we performed complementation and gene overexpression of *crf4-3* in the *crf4-3*^KO^ strain. When *crf4-3* gene was reintroduced into the *crf4-3*^KO^ strain, the sensitivity of the knockout strain to ketoconazole (Fig. 3A and B) and the transcriptional response of *erg11* to ketoconazole were restored to the wild-type levels (Fig. 3C). We also tested the sensitivity of these strains to other azole drugs, such as voriconazole and itraconazole, and obtained results similar to those with ketoconazole (Fig. 3A and B). These results proved further that Crf4-3 is involved in the transcriptional response of *erg11* to azole drugs and affects the sensitivity of *N. crassa* to azole drugs. Overexpression of *crf4-3* (about 5-fold) could also restore the phenotypes of the *crf4-3* knockout strain without any increases in the resistance of the strain to azoles or the transcriptional response level of *erg11* to ketoconazole (Fig. 3A through C; Fig. S3).

Crf4-3 was identified through DNA-pull down assay, an *in vitro* technique that may inadvertently capture proteins with non-specific DNA binding affinity. To validate the *in vivo* binding of Crf4-3 to the *erg11* promoter, we employed a ChIP-qPCR assay to investigate the interaction between GFP-tagged Crf4-3 and the *erg11* promoter region. As expected, we observed a significant enrichment of Crf4-3 at *erg11* promoter, with a 16-fold increase under normal conditions (Fig. 3D). Notably, this enrichment dramatically escalated to approximately 23-fold in the presence of ketoconazole (Fig. 3D), signifying the enhanced recruitment of the Crf4-3 protein to the *erg11* promoter under azole stress. Surprisingly, we also detected enrichment of Crf4-3 within the *erg11* coding sequence, which intensified following azole exposure (Fig. 3D). These observations imply that Crf4-3 can directly interact with both the *erg11* promoter and its gene *in vivo*, thereby modulating the transcriptional response of *erg11* to azoles.

### Crf4-3 specifically affects the sensitivity of *N. crassa* to azole drugs

To figure out whether Crf4-3 is a general stress response regulator, we examined the effects of *crf4-3* knockout on sensitivity to various stresses. These include the sterol biosynthesis inhibitor terbinafine targeting ERG1, the sterol biosynthesis inhibitors amorolfine targeting ERG24 and ERG2, the sphingolipid biosynthesis inhibitor myriocin, the mitochondrial respiratory chain complex I inhibitor rotenone, the oxidative stress inducers menadione and hydrogen peroxide, the membrane damage inducer sodium dodecyl sulfate (SDS), the endoplasmic reticulum stress inducer dithiothreitol (DTT), the osmotic stress inducer NaCl, and heat stress. All the stresses inhibited the growth of the wild-type *N. crassa* strain to varying degrees, but the *crf4-3* knockout did not result in higher inhibition (Fig. S4), especially with terbinafine and amorolfine, which are also ergosterol biosynthesis inhibitors like azoles. Therefore, Crf4-3 is specifically involved in the transcriptional response of *N. crassa* to azole stress.

### Crf4-3 is a protein stably present in the nucleus

**Fig 4:**
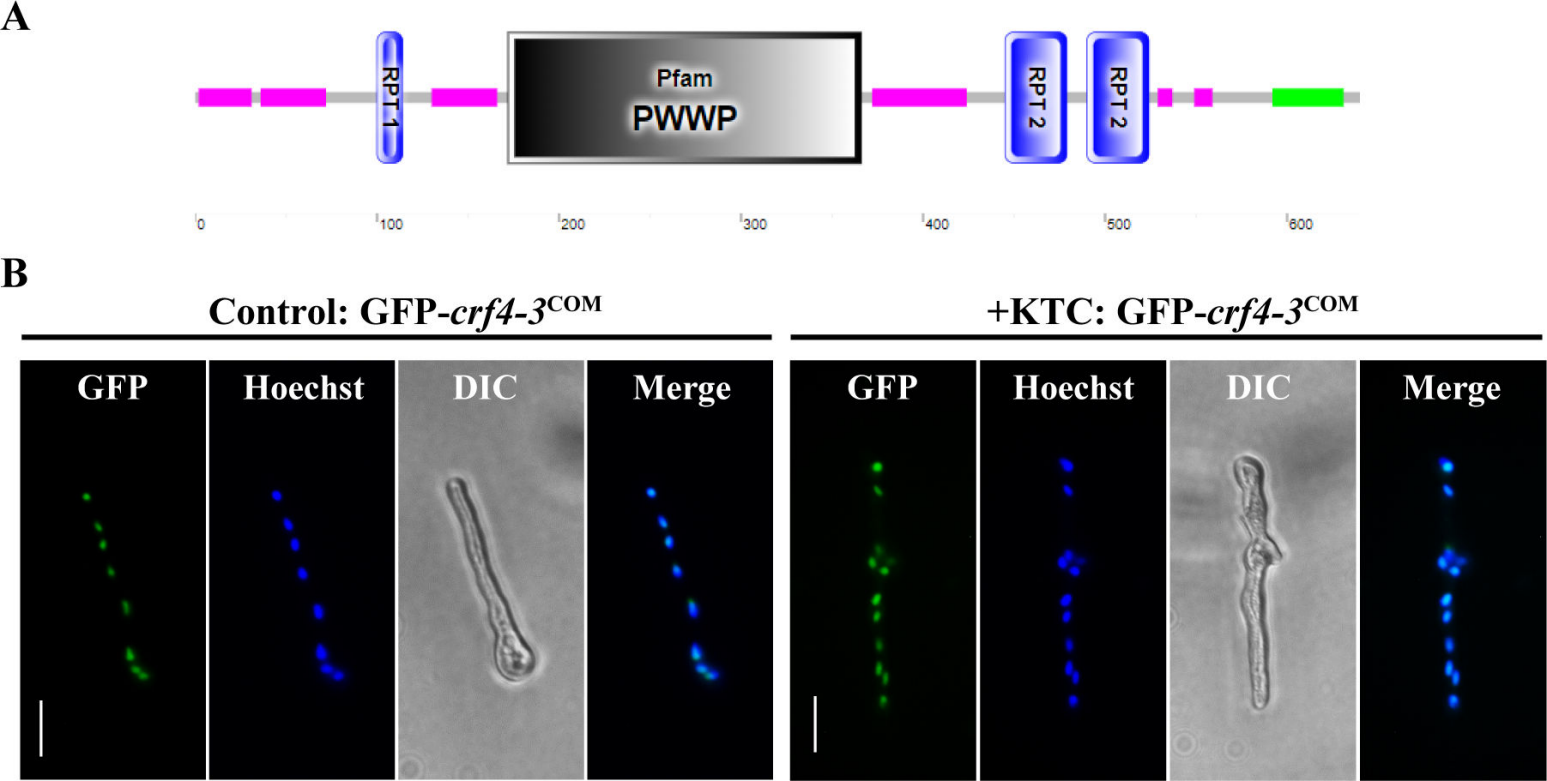
Crf4-3 is a nuclear protein. (**A**) The domain architecture of Crf4-3 according to the SMART protein database (http://smart.embl-heidelberg.de). (**B**) GFP-Crf4-3 is localized to the nucleus under both normal growth and ketoconazole treatment conditions. The conidia suspension (2 × 10^6^ spores/mL) of GFP-*crf4-3*^COM^ strain was germinated at 28°C and 200 rpm for 3 h and then treated with or without 1.5 µg/mL ketoconazole (KTC) for 3 h. GFP fluoresces were observed under a Zeiss fluorescent microscope. Bar = 10 µm.

Crf4-3 is predicted as a PWWP domain-containing protein (Fig. 4A). This kind of protein typically functions as a nuclear protein to regulate numerous biological processes, including transcription (42–44). To determine whether Crf4-3 also functions as a nuclear protein, we constructed a GFP-tagged Crf4-3 under the control of its promoter and observed its subcellular localization. Under normal growth conditions, Crf4-3 was localized in the nucleus (Fig. 4B), indicating that it is a nuclear protein. Under ketoconazole treatment, Crf4-3 remained localized in the nucleus (Fig. 4B), suggesting that azole drug stress does not affect the localization of this protein. These results indicate that Crf4-3 is a nuclear protein.

### Crf4-3 modulates azole resistance in a manner independent of the ISWI complex

Crf4-3 is co-purified with the ISWI complex (45). To investigate whether Crf4-3 modulates azole resistance as part of the ISWI complex, we assessed the drug sensitivity of mutations lacking the core component of the ISWI complex *iswi* (NCU03875), *iaf-1* (NCU00412), and *iaf-2* (NCU09388). Among them, NCU00412 and NCU09388 were identified as ISWI-interacting proteins in *N. crassa* and named ISWI accessory factors 1 and 2 (IAF-1 and IAF-2), respectively (45, 46). However, unlike the *crf4-3*^KO^ strain, deletion of each core component of the ISWI complex did not change their sensitivity to ketoconazole (Fig. S5), suggesting that the ability of Crf4-3 to regulate azole resistance is independent of the ISWI complex.

### Crf4-3 is involved in the transcriptional response to azole stress and positively regulates the expression of ergosterol biosynthesis genes

The transcriptional response of *erg11* to ketoconazole was significantly affected by *crf4-3* knockout, suggesting that Crf4-3 may regulate the transcriptional response of fungi to azole stress, which may be a key reason for its impact on azole drug sensitivity. Therefore, we analyzed the impact of *crf4-3* knockout on the whole-genome transcriptional profile before and after azole treatment by RNA sequencing (RNA-seq). A total of 9,421 genes were detected. After removing low abundance genes with FPKM (fragments per kilobase of transcript per million mapped reads) values less than five across all groups, 7,027 genes were subjected to further analysis (Data Set S2).

In the wild-type strain, ketoconazole treatment resulted in the upregulation of 1,161 (screening criteria: log_2_Fold change_WT-KTC/WT-Control ≥1; qvalue＜0.05) genes and the downregulation of 1,093 genes (screening criteria: log_2_Fold change_WT-KTC/WT-Control≤−1; qvalue＜0.05). After *crf4-3* knockout, the transcriptional upregulation of 129 genes (11.1%) in response to ketoconazole was weakened (screening criteria: log_2_Fold change_WT-KTC/WT-Control ≥1 and Fold change_*crf4-3*^KO^-KTC/WT-KTC＜0.67; qvalue＜0.05), and the transcriptional downregulation of 110 genes (10.1%) was weakened (screening criteria: log_2_Fold change_WT-KTC/WT-Control≤−1 and Fold change_*crf4-3*^KO^-KTC/WT-KTC＞1.5; qvalue＜0.05). These results indicate that Crf4-3 regulates the transcriptional response of *N. crassa* to azole drug stress.

**Fig 5:**
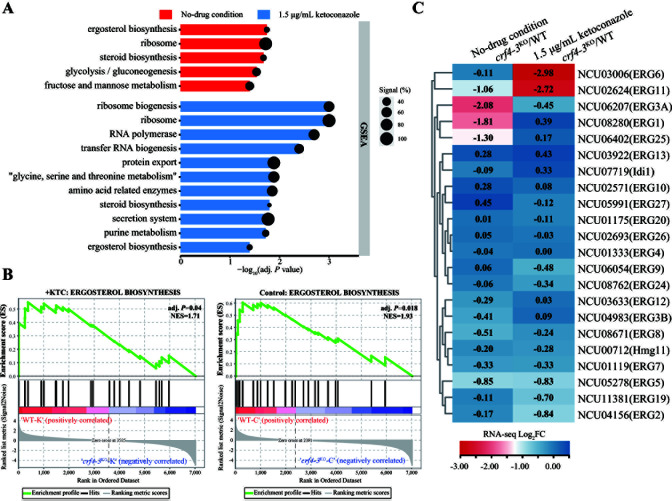
Crf4-3 positively regulates the expression of ergosterol biosynthesis genes. (**A**) GSEA of gene expression profiles of *crf4-3*^KO^ and wild-type strains under normal growth conditions and ketoconazole treatment conditions. (**B**) Enrichment plot of the ergosterol biosynthesis pathway genes after *crf4-3* knockout under normal growth conditions and ketoconazole treatment conditions. The enriched pathway with a NES greater than one and an adjusted *P*-value (adj. *P*) less than 0.05 was considered significant. (**C**) Changes in gene expression of ergosterol biosynthesis genes after *crf4-3* knockout under normal growth and ketoconazole treatment conditions. The values labeled in the heatmap represent the logarithm of the fold change in FPKM values according to RNA-seq.

Further gene set enrichment analysis (GSEA) revealed that the ergosterol biosynthesis pathway was significantly enriched after *crf4-3* knockout in the settings of both normal growth condition and ketoconazole treatment condition (screening criteria: normalized enrichment score [NES] greater than one and adjusted *P*-value [adj. *P*] less than 0.05) (Fig. 5A and B; Data Set S3). We subsequently focused on genes related to ergosterol biosynthesis. We found that under normal growth conditions, the knockout of *crf4-3* reduced the basal expression of *erg1*, *erg11*, *erg25*, and *erg3A* (Fig. 5C). Under ketoconazole treatment, the knockout of *crf4-3* significantly affected the inducible expression of *erg11*, which was in line with the results of qRT-PCR (Fig. 5C and 3C). Also, the transcriptional response of *erg6* was attenuated in the *crf4-3* knockout strain (Fig. 5C). Above all, our results indicate that Crf4-3 positively regulates the expression of ergosterol biosynthesis genes under both normal growth and azole stress conditions.

### Crf4-3 homologs are widely present in *Pezizomycotina* fungi

**Fig 6:**
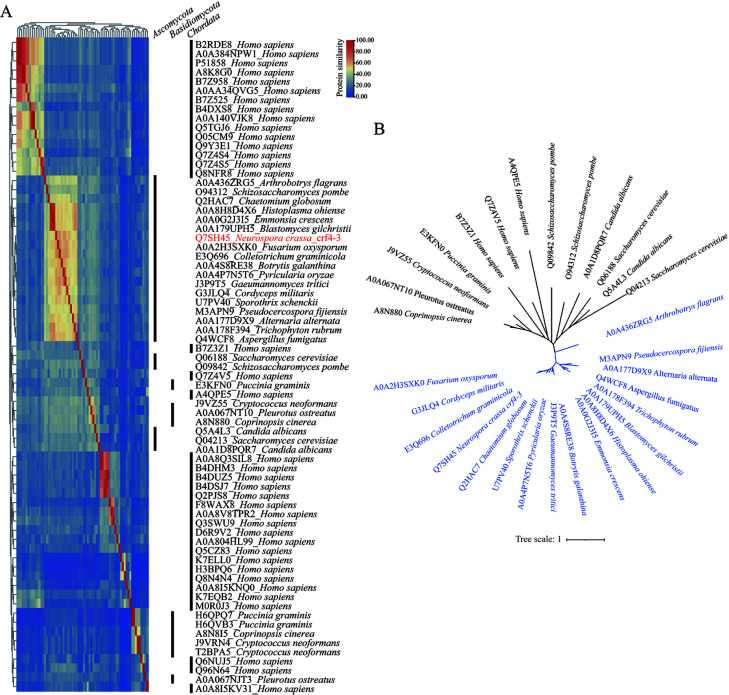
Crf4-3 homologs are widely present in *Pezizomycotina* fungi. (**A**) Protein pairwise similarity matrix analysis. The amino acid sequences used for similarity analysis were the PWWP domain-containing proteins in representative fungal species and proteins containing a single PWWP domain in *Homo sapiens*. The analysis was performed using TBtools v2.106. The PWWP domain-containing protein Crf4-3 in *N. crassa* was highlighted in red. (**B**) Phylogenetic tree of PWWP domain-containing proteins exhibiting high sequence similarity with Crf4-3. The amino acid sequences of PWWP domain-containing proteins from *Pezizomycotina* were highlighted in blue. PhyloSuite 2 was used to conduct, manage, and streamline the phylogenic analyses with the help of several plug-in programs. iTOL was used for tree visualization and modification.

PWWP domain-containing proteins are widely present in eukaryotes (47). They regulate various biological processes in combination with other functional domains in mammals and plants (48–50). However, only one protein consisting of just a PWWP domain was found in most fungi, especially in *Ascomycota*. To address the relationships between these proteins in fungi, as well as their potential homologs in other eukaryotes, we collected all the PWWP domain-containing proteins in several representative fungi, as well as proteins with only a PWWP domain in humans, and constructed a protein pairwise similarity matrix. As shown, although Crf4-3 clustered with fungal PWWP domain-containing proteins, it compactly clustered with proteins from *Pezizomycotina* fungi (Fig. 6A). This is consistent with our BLAST (Basic Local Alignment Search Tool) searches, in which Crf4-3 homologs are only found in filamentous fungi of the *Ascomycota* phylum but with low sequence identity in *Saccharomyces cerevisiae* (Q04213; 39.47% identity and 5% query coverage) and *Homo sapiens* (Q7Z4V5; 24.1% identity and 12% query coverage). No Crf4-3 homologs were found by BLAST in *Arabidopsis thaliana*, *Drosophila melanogaster*, and *Caenorhabditis elegans*. To further characterize their phylogenic relationship, proteins clustered together with Crf4-3 were chosen for phylogenic tree construction. In the unrooted tree, proteins from *Pezizomycotina* formed a separated clade distinct from that of *Saccharomycotina* and *Basidomycota*, as well as *Homo sapiens* (Fig. 6B). Additionally, multiple sequence alignment analysis showed that the amino acids of Crf4-3 homologs from *Pezizomycotina* are highly conserved at many positions (Fig. S6). These indicate that although PWWP domain-containing proteins are widely present in eukaryotes, the Crf4-3 homolog might be a conserved *Pezizomycotina* protein.

### The homolog of Crf4-3 in *A. fumigatus* affects azole sensitivity and *erg11* gene expression

Given the role of Crf4-3 in fungal resistance to azole drugs, verifying this function in other fungi, especially some pathogenic fungi, is of great significance. Therefore, we selected *A. fumigatus*, an important human pathogenic fungus evolutionarily distant from *N. crassa*, for further study. In *A. fumigatus*, the protein encoded by AFUA_8G04570 shared a high similarity with Crf4-3 (44.85% identity; 60% query coverage) and was named *A. fumigatus* CrfA in this study.

**Fig 7:**
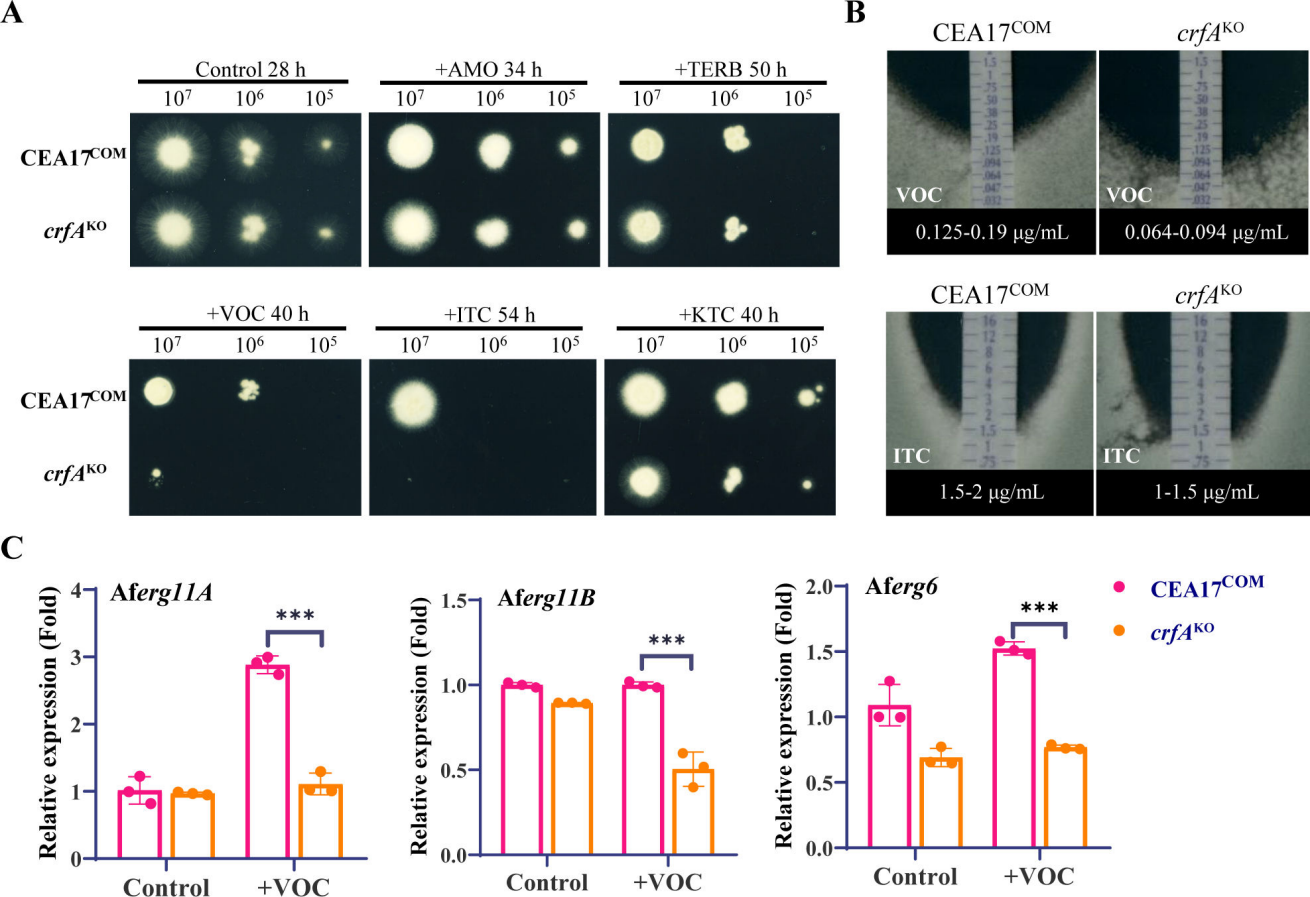
The homologous protein of Crf4-3 in *A. fumigatus* has conserved functions. (**A**) Sensitivity tests of CEA17^COM^ and *crfA*^KO^ strains to different sterol biosynthesis inhibitors. Two microliters of serial 10-fold dilutions of a conidial suspension was inoculated onto complete medium (CM) plates with or without antifungal compounds. All plates were incubated at 37°C in the dark. VRC: voriconazole (0.25 µg/mL); ITC: itraconazole (0.4 µg/mL); KTC: ketoconazole (2.5 µg/mL); AMO: amorolfine (10 µg/mL); and TERB: terbinafine (0.3 µg/mL). (**B**) The MICs of VRC and ITC for the CEA17^COM^ and *crfA*^KO^ strains were determined by the E-test method. Conidia of *A. fumigatus* strains were equally inoculated and uniformly spread onto the CM plate. E-test strips were then placed in the middle of the plates. All plates were cultured at 37°C for 48 h. (**C**) Gene expression detection of *erg11A*, *erg11B*, and *erg6* in CEA17^COM^ and *crfA*^KO^ strains treated with 0.25 µg/mL voriconazole for 12 h. Gene expression was detected using qRT-PCR. Expression levels were calculated using the 2^–ΔΔCT^ method with the expression of the *actin* gene as an internal reference. The transcription level of each gene in the CEA17^COM^ strain without treatment was normalized to 1. The levels of significance for differences between the two strains are indicated by asterisks (*, *P* < 0.05; **, *P* < 0.01; ***, *P* < 0.001). Error bars indicate standard deviations, *n* = 3.

To investigate the function of CrfA, we constructed a *crfA* knockout strain and analyzed its sensitivity to azole drugs. On plates without antifungal drugs, the *crfA*^KO^ strain showed no significant difference in colony morphology compared with the complemented strain CEA17^COM^ (Fig. 7A). However, on plates containing 0.25 µg/mL voriconazole, 0.4 µg/mL itraconazole, or 2.5 µg/mL ketoconazole, the colony size of the *crfA*^KO^ strain was significantly smaller than that of the CEA17^COM^ strain, especially with voriconazole and itraconazole (Fig. 7A). Furthermore, E-test experiments showed that the voriconazole MICvalue of the *crfA*^KO^ strain (0.064–0.094 μg/mL) was about half that of the CEA17^COM^ strain (0.125–0.19 μg/mL), and the itraconazole MIC value of the *crfA*^KO^ strain (1–1.5 μg/mL) was also lower than that of the CEA17^COM^ strain (1.5–2 μg/mL) (Fig. 7B). These results indicate that *crfA* knockout also leads to increased sensitivity of *A. fumigatus* to azoles. Interestingly, *crfA* knockout did not affect the sensitivity of *A. fumigatus* to two other sterol biosynthesis inhibitors, amorolfine and terbinafine, similar to *N. crassa* (Fig. 7A; Fig. S4).

In *N. crassa*, the *crf4-3* knockout affects the transcriptional response of the ergosterol biosynthesis genes *erg11* and *erg6* to ketoconazole (Fig. 5C). We thus verified whether CrfA also affects the transcriptional response of *erg11* and *erg6* to azole treatment. As expected, CEA17^COM^ strain upregulated the expression level of *erg11A* by about three times under voriconazole stress, but after *crfA* knockout, *erg11A* had no response to azole treatment at the transcriptional level (Fig. 7C). Although *erg11B* was not upregulated under voriconazole treatment, its expression level significantly decreased under voriconazole treatment after *crfA* knockout (Fig. 7C). Similar to *erg11A*, the *erg6* gene lost its transcriptional response to voriconazole after *crfA* knockout (Fig. 7C). These results indicate that in *A. fumigatus*, CrfA also regulates the transcriptional response of the ergosterol biosynthesis genes *erg11* and *erg6*, thereby affecting its sensitivity to azoles.

## DISCUSSION

Although other mechanisms contribute, transcriptional control of pivotal genes, such as *erg11*, and those encoding efflux pumps stand as the primary driver of azole resistance. Deciphering the underlying mechanisms holds significant value in comprehending antifungal resistance and in devising innovative strategies to counteract it. Over the past few decades, considerable effort has been dedicated to identifying transcription factors that participate in regulating the response to azole stress, thereby enhancing our understanding of azole resistance (51–53). However, the involvement of other proteins in this process, particularly those that are more conserved and potentially druggable, remains less understood. In this study, we utilized a DNA pulldown assay targeting key promoter region to identify potential direct regulators of *erg11*, the gene encoding azole target. We unexpectedly uncovered a PWWP domain-containing protein, Crf4-3 (NCU02684), as a novel regulator of azole resistance. The deletion of *crf4-3* led to increased sensitivity specifically to azoles and altered the transcriptional response of *erg11* to azoles. Interestingly, Crf4-3 operates as a nuclear protein, modulating not only the responses of *erg11* and *erg6* to azoles but also the baseline expression of *erg1*, *erg11*, *erg25*, and *erg3A*, suggesting its role in governing sterol homeostasis. More importantly, Crf4-3 is highly conserved among *Pezizomycotina*, which encompasses numerous fungi pathogenic to humans and plants. Deletion of *crfA* in *A. fumigatus*, a pathogenic fungus evolutionarily distant from *N. crassa*, also increased sensitivity to azoles and diminished the transcriptional response of *erg11* to voriconazole. Therefore, the regulation of the transcriptional response to azoles by Crf4-3 may be a general mechanism enabling filamentous fungi in *Pezizomycotina* to adapt to azole stress. Above all, our study characterizes a novel conserved protein that is required for azole resistance and uncovers its role in transcriptional regulation of key genes like *erg11* under azole stress, offering fresh insights into mechanisms of azole resistance and providing a potential target for the development of drugs that combat azole resistance.

Crf4-3 is a PWWP domain-containing protein. The PWWP domain, named after the conserved Pro-Trp-Trp-Pro sequence motif, is a small domain consisting of 100–150 amino acids. It binds both DNA and lysine-methylated histone and plays important roles in biological processes such as transcription regulation and DNA methylation and repair (49, 54, 55). The proteins containing this domain are extensively studied in mammals and plants; however, its functional roles in fungi, beyond budding yeast, remain largely enigmatic. We have characterized Crf4-3, the sole PWWP domain-containing protein in *N. crassa*, as a key regulator of azole resistance. This particular phenotype was also observed in *A. fumigatus* within the scope of our study. Additionally, deletion of Ioc4p, the sole PWWP protein in budding yeast, also resulted in azole hypersensitivity (56). This indicates that fungal PWWP proteins may play a conserved role in the regulation of azole resistance. Remarkably, Crf4-3 is selectively involved in resistance to azoles but does not play a role in response to other stressors examined in our study, including two distinct sterol biosynthesis inhibitors that do not target ERG11. This suggests that the protein may fulfill a highly specialized function within fungi.

Since Crf4-3 functions as a nuclear protein and binds not only *erg11* promoters but also the *erg11* gene, it may not be a transcription factor. Crf4-3 is postulated to be the homolog of *S. cerevisiae* Ioc4p, a member of the Isw1b chromatin remodeling complex (57), although the sequence similarity is low. Interestingly, research in *N. crassa* has also revealed that Crf4-3 can be co-purified with the ISWI proteins (45). Chromatin assembly has been reported to be associated with antifungal resistance in multiple pathogenic fungi (58–61). It is plausible that Crf4-3 influences azole resistance in concert with the ISWI complex through the modulation of chromatin remodeling. However, the deletion of the core component of the ISWI complex did not alter azole susceptibility in *N. crassa*. In budding yeast, the deletion of Isw1p resulted in decreased sensitivity to one of the five azoles tested, whereas the deletion of Ioc4p induced heightened sensitivity to four of the five azoles (56). These findings highlight a distinction between PWWP proteins and the ISWI chromatin remodeling complex, suggesting that Crf4-3 modulates azole resistance via alternative mechanisms.

The transcription factors directly involved in regulating sterol biosynthesis have already been identified, such as Upc2p in *S. cerevisiae*, SrbA and AtrR in *A. fumigatus,* and FgSR in *F. graminearum* (24, 41, 62, 63). Although Crf4-3 is likely not a transcription factor, it might collaborate with these transcription factors. It is proposed that the PWWP domain is a histone mark reader and functions as a platform for protein-protein interactions (42). A plausible mechanism is that Crf4-3 participates in interactions with transcription factors and/or components of chromatin remodeling and transcription machinery complex, thereby regulating their binding to specific genomic loci or their interactions. Therefore, it would be not surprising that overexpression of Crf4-3 has little effect on *erg11* expression and azole resistance. We interestingly found that the binding sites for FgSR and Upc2p are among the sites that resemble the critical region of the *N. crassa erg11* promoter, which were used to identify Crf4-3, giving an implication of Crf4-3 and transcription factor interaction. Unfortunately, we did not identify any key transcription factors in our DNA pulldown assay due to unknown reasons. Also, none of the known transcription factors required for azole resistance were among the proteins interacting with Crf4-3 identified in *N. crassa* (45). Furthermore, *N. crassa* lacks homologs of Upc2p, and as of now, a mutant for the FgSR homolog is not readily available. Thus, the precise mechanism by which Crf4-3 governs azole resistance warrants additional investigation for full elucidation.

More interestingly, it has been observed that Crf4-3 selectively governs the transcriptional response of *erg11* and *erg6* to azoles, influencing susceptibility to azoles exclusively. As reported in yeast and *Candida albicans*, when sterol biosynthesis is inhibited, transcriptions of a suite of *erg* genes including *erg11*, *erg6*, *erg24*, and others are upregulated collectively through ergosterol feedback regulation (64–66). This suggests that these *erg* genes are co-regulated by a common mechanism in response to the depletion of ergosterol. However, in our study, although azole treatment results in the upregulation of multiple *erg* genes, only the responses of *erg11* and *erg6* are modulated by Crf4-3. Therefore, the regulatory mechanisms governing different *erg* genes may exhibit distinct characteristics in fungi like *N. crassa* and *A. fumigatus*. This notion is further supported by previous reports, indicating that *erg* genes exhibit differential responses to various types of sterol biosynthesis inhibitors (67–69). Nevertheless, the differential responses of *erg* genes in fungi remain poorly understood and necessitate further investigation.

In summary, this study identifies a novel regulatory protein, Crf4-3, which is involved in regulating the transcriptional responses of ergosterol biosynthesis genes to azoles. This is also the first report in that a PWWP domain-containing protein contributes to the mechanism behind fungal azole resistance. Crf4-3 is particularly conserved in filamentous fungi of the subphylum *Pezizomycotina* and plays similar roles in the human pathogenic fungus *A. fumigatus*, as well as in budding yeast, thus providing new insight into the regulation of azole stress response. More importantly, small molecules targeting PWWP domains have been reported in human studies, suggesting that this domain is a feasible and accessible target for drug design. As a protein equipped with a PWWP domain, Crf4-3 presents itself as a promising target for the development of fungal-specific drugs capable of addressing azole resistance.

## MATERIALS AND METHODS

### Strains and growth conditions

All strains used in this study are listed in Table S1.

All *N. crassa* strains were cultured on Vogel’s medium (with 2% sucrose for slants and with 2% glucose for plates and liquid culture) and solidified with 1.5% agar if required. Sorbitol medium (Vogel’s medium with 2% sorbitol, 0.05% fructose, and 0.05% glucose) was used for electroporation. Antifungal drugs were added as needed. All cultures were grown at 28°C.

All *A. fumigatus* strains were cultured on CM (70), solidified with 1.5% agar if required. Antifungal drugs were added as needed. All cultures were grown at 37°C.

### Construction of *gfp* reporter system

The promoter of *N. crassa erg11* was analyzed by partial deletion as shown in the schematic diagram (Fig. 1A) using the *gfp* gene as a reporter. The reporter system was constructed following procedures to target gene constructs to the *his-3* locus in *N. crassa* (71, 72). Briefly, fragments containing different *erg11* promoter regions were amplified, respectively, with corresponding primer pairs listed in Table S2. These fragments along with the *gfp* coding sequence (741 bp) and the *trpC* terminator (774 bp) were then inserted into plasmid pBM61 digested by EcoRI through a one-step cloning kit (ClonExpress Ultra One Step Cloning Kit; Vazyme, China), generating a series of *gfp* reporter plasmids. These plasmids were then introduced into the histidine-deficient strain (*his-3*^–^) by electroporation. Positive transformants were selected on plates without histidine and verified by PCR. All primers used for construction and validation are listed in Table S2.

The FIMO analysis was conducted using MEME Suite 5.5.5 (73). The 1 kb promoters of *erg11* orthologous genes from 21 representative fungi were used for the FIMO analysis (Data set S1). The *P*-value of a motif occurrence is defined as the probability of a random sequence of the same length as the motif matching that position of the sequence with as good or better a score.

### Deletion of *crf4-3* in *N. crassa*

Deletion of *crf4-3* in *N. crassa* was performed with the homologous recombination method, using hygromycin B resistance gene *hph* as the selective marker. Briefly, the promoter of the *crf4-3* gene (667 bp) was amplified using the primers *crf4-3*ko-up-F/R, the *hph* gene (1450 bp) was amplified using the primers *crf4-3*ko-*hph*-F/R, the terminator of the *crf4-3* gene (721 bp) was amplified using the primers *crf4-3*ko-down-F/R, and the plasmid backbone fragment was amplified using the primers pCSN43-kpni-hind-F/R. The obtained fragments were then assembled and resulted in the plasmid pCSN-KO-*crf4-3* using a one-step cloning kit (ClonExpress Ultra One Step Cloning Kit; Vazyme, China). The knockout fragment, comprising the promoter of the *crf4-3* gene, the *hph* coding sequence, and the terminator of the *crf4-3* gene, was amplified with primers T7/3 and purified via gel extraction. The purified fragment was introduced into *N. crassa* N85 (*bd Ku70^RIP^*) strain via electroporation (74). Transformants were screened on Vogel’s slants containing 150 µg/mL hygromycin B and verified by PCR. Subsequently, the obtained knockout mutation was crossed with the *N. crassa* wild-type strain (FGSC#2225) to eliminate the genetic background of *bd* and *Ku70^RIP^* mutations. All primers used to construct and verify the *crf4-3* knockout strain are listed in Table S2.

### Complementation and overexpression of *crf4-3* in *N. crassa crf4-3*^KO^ strain

The plasmid pCB1532 containing the chlorimuron ethyl resistance gene was used to construct plasmids pCOM-*crf4-3*, pCOM-GFP-crf4-3, and pOE-GFP-*crf4-3*, which are used for the complementation and overexpression of *crf4-3* in the *crf4-3*^KO^ strain. Briefly, the 4,742 bp fragment, containing the coding sequence, the upstream region, and the downstream region of *crf4-3*, was amplified, purified, and inserted into plasmid pCB1532 digested by XbaI and KpnI through a one-step cloning kit (ClonExpress Ultra One Step Cloning Kit; Vazyme, China), resulting in plasmid pCOM-*crf4-3*. Likewise, the 1,082 bp promoter of the *crf4-3*, 737 bp coding sequence of the *gfp*, 2,377 bp coding sequence of the *crf4-3*, and 1,814 bp terminator of the *crf4-3* were amplified, purified, and inserted into the plasmid pCB1532 digested by XbaI and KpnI through a one-step cloning kit, resulting in plasmid pCOM-GFP-*crf4-3*. Similarly, the 923 bp promoter of the *tef-1*, 757 bp coding sequence of the *gfp*, 2,417 bp coding sequence of the *crf4-3*, and 774 bp terminator of the *trpC* were amplified, purified, and ordered inserted into the plasmid pCB1532 digested by XbaI and KpnI through a one-step cloning kit, resulting in the plasmid pOE-GFP-*crf4-3*.

The pCOM-*crf4-3*, pCOM-GFP-*crf4-3*, and pOE-GFP-*crf4-3* were introduced into the *crf4-3*^KO^ strain using the electroporation method, as detailed in a previous investigation (69). Positive transformants were selected on a medium supplemented with 20 µg/mL chlorimuron ethyl and were further confirmed by PCR. All primers used for construction and validation were listed in Table S2.

### RNA extraction and qRT-PCR analysis

For *N. crassa*, fresh conidia were inoculated onto plates containing liquid Vogel’s medium and grown for 30 h. The obtained mycelia film was then torn into small pieces. Twelve 1–2 mm² mycelial pieces were inoculated into 100 mL Vogel’s liquid medium and grown at 28°C with 200 rpm shaking for 12 h. The experimental group was treated with ketoconazole (1.5 µg/mL) for another 12 h, whereas the control group was grown for another 12 h with equal volume dimethyl sulfoxide (DMSO). The collected mycelia were ground into fine powder in liquid nitrogen, and RNA was extracted using the standard TRIzol (Invitrogen, United States) method. Two micrograms of total RNA was reverse-transcribed into cDNA using the cDNA synthesis kit (HiScript III All-in-one RT SuperMix Perfect for qPCR; Vazyme, China). qRT-PCR was performed on a CFX96 multicolor real-time PCR detection system (Bio-Rad, United States) using SYBR (ChamQ Universal SYBR qPCR Master Mix; Vazyme, China). Gene expression levels were calculated using the 2^−△△CT^ method. The expression level of each gene was normalized by the expression level of *β-tubulin*. The experiments were set up with three replicates. Error bars represent the standard deviation of the mean.

For *A. fumigatus*, 10^6^ conidia were inoculated into 100 mL CM liquid medium and grown at 37°C with 200 rpm shaking for 12 h. The experimental group was treated with voriconazole (0.25 µg/mL) for an additional 12 h, whereas the control group was grown for an additional 12 h with equal volume DMSO. The mycelia were then collected and stored at −80°C. RNA extraction and qRT-PCR analysis were performed as described above. In *A. fumigatus*, the expression level of each gene was normalized to the expression level of *actin*.

### RNA-seq and data analysis

Transcriptomic profiles of the *N. crassa* wild-type and *crf4-3*^KO^ strains, either treated with KTC or left untreated, were analyzed by high-throughput RNA sequencing (RNA-seq). Sample preparation and total RNA extraction were performed as described above. Each treatment was set up with three biological replicates. Total RNA was subjected to the BGIseq-500RS platform for RNA-seq and data processing. The reference genome of *N. crassa* was obtained from FungiDB (https://fungidb.org/common/downloads/Current_Release/NcrassaOR74A/). Genes with FPKM values less than five in all samples were considered low-abundance genes and were removed from subsequent analysis. Differentially expressed genes were identified based on expression changes > 2 or <0.5 between two samples, with a q-value (adjusted *P*-value) <0.05.

GSEA was performed using GSEA 4.3.2. The gene expression data of the *N. crassa* wild-type and *crf4-3*^KO^ strains were analyzed in the GSEA software using 1,000 permutations to determine enriched pathways. The pathways with a NES greater than one and an adjusted *P*-value (adj. *P*) less than 0.05 were considered significant.

### Stress sensitivity assay

The plate drug sensitivity test for *N. crassa* was conducted following the established procedure (75). Briefly, conidia were collected and washed from 7-day-old *N. crassa* slants using sterile water. Two milliliters of conidial suspension (2 × 10^6^ conidia/mL) was inoculated onto Vogel’s medium plates with or without compounds and incubated at 28°C. To enhance the observability and precision of data, as well as to facilitate a more accurate comparison of *N. crassa* growth under inhibitory conditions, an extended incubation period was employed, with the control condition set at approximately 29 h. The compounds used included 1.5 µg/mL ketoconazole (Sigma-Aldrich, United States), 0.5 µg/mL voriconazole (Selleck, United States), 20 µg/mL Itraconazole (MedChemExpress, United States), 2.5 µg/mL terbinafine (J&K, China), 0.375 µg/mL amorolfine (TCI, Japan), 20 ng/mL myriocin (Enzo, United States), 2 mM dithiothreitol (Sigma-Aldrich, United States), 80 µg/mL rotenone (Sigma-Aldrich, United States), and 0.5 µg/mL menadione (Selleck, United States).

The plate drug sensitivity test for *A. fumigatus* was conducted according to previous studies (25). Briefly, conidia were collected from 3-day-old *A. fumigatus* cultures using 0.02% Tween 20, washed three times with sterile water, and diluted to 10^7^, 10^6^, and 10^5^ conidia/mL. Two milliliters of conidial suspension was inoculated onto CM plates with or without antifungal drugs and incubated at 37°C. The antifungal drugs used included 2.5 µg/mL ketoconazole (Sigma-Aldrich, United States), 0.25 µg/mL voriconazole (Selleck, United States), 0.4 µg/mL Itraconazole (MedChemExpress, United States), 0.3 µg/mL terbinafine (J&K, China), and 10 µg/mL amorolfine (TCI, Japan).

### DNA-pulldown assay

The pulldown assay was performed to isolate *N. crassa* proteins capable of binding to the key sequence of *erg11* promoter. A 40 bp fragment containing the key promoter region in its middle was labeled with biotin at both ends and used as the probe for the pulldown assay. The assays were slightly modified based on previous studies (76). Briefly, mycelial samples were prepared following RNA extraction protocols. Nuclear proteins were extracted from mycelial samples, treated with DNase, and used for the pulldown assay (77). For pulldown, streptavidin magnetic beads (MedChemExpress, United States) (40 µL/sample) were blocked in 1× bind buffer (25 mM HEPES-KOH, pH 7.9; 20 mM KCl, 10% vol/vol glycerol; 1 mM DTT; 0.2 mM EDTA; 5 mM MgCl_2_; 20 µM ZnCl_2_; and phosphatase and protease inhibitors added before use) with 0.5 wt% bovine serum albumin (BSA) and 10 µM random primer. An appropriate amount (2 µg/1,000 bp) of biotin-labeled DNA probe was then pre-bound to the Streptavidin magnetic beads. The pulldown assay mixture comprised 300 µL of 2× binding buffer, 3 µL of Poly (dI) (at a concentration of 1 µg/µL), 60 µg of nuclear protein extract, 1 µL of 1 M DTT, 6 µL of 0.5 M EDTA, 3 µL of 0.5 M EGTA, and 1× protease inhibitor cocktail. Double-distilled water was added to adjust the final volume to 600 µL. A mock reaction containing oligonucleotide-free streptavidin magnetic beads was used as a control. The mixture was then incubated at 4°C for 1 h. After incubation, the beads were washed three times with wash buffer (1× bind buffer with 10 mM NaCl), and the enriched proteins were eluted with elution buffer (1× bind buffer with 500 mM NaCl), followed by boiling for 10 min. The proteins were separated on 12% SDS-PAGE and silver-stained. Protein bands present in the reaction but absent in the mock reaction were excised from the gel, digested with trypsin, and analyzed by mass spectrometry.

### Chromatin immunoprecipitation (ChIP)-qPCR analysis

ChIP was performed following a previously described protocol with some modifications (78). Briefly, mycelia grown in liquid Vogel’s medium were cross-linked with 1% formaldehyde for 15 min, and the reaction was halted with 125 mM glycine for 5 min. The collected mycelia were ground into a fine powder with liquid nitrogen and suspended in the lysis buffer containing protease inhibitors (Beyotime, China). DNA was sheared into 100–800 bp fragments under ultrasonic treatment. Immunoprecipitation was conducted using a rabbit polyclonal anti-GFP antibody (ab290, Abcam, UK; 1:500 dilution) and protein G magnetic beads (P2106, Beyotime, China). As a control, rabbit IgG magnetic beads (P2173, Beyotime, China) were used for immunoprecipitation. The beads were sequentially washed with low salt buffer, high salt buffer, LiCl buffer, and TE buffer. DNA was then eluted from the magnetic beads and precipitated by glycogen after de-crosslinking, RNase A treatment, and protease K digestion. The obtained DNA solution was analyzed by quantitative PCR using the primers provided in Table S2.

### Fluorescent microscopic observation of GFP-tagged Crf4-3 strain

Spore suspension with a final concentration of 2 × 10^6^ conidia/mL for each strain was incubated at 28°C with shaking at 200 rpm for 3–4 h to germinate. The germinated spores were then treated with 1.5 µg/mL ketoconazole for 3 h, whereas those treated with an equal volume of DMSO for 3 h were used as a control. After that, the mycelia were stained with 1× Hoechst 33342 (Beyotime, China) for 10 min and then washed three times with liquid Vogel’s medium before fluorescence microscopy observation. If necessary, an Antifade Mounting Medium (Beyotime, China) is used to reduce fluorescence quenching.

### Construction of protein pairwise similarity matrix and phylogenetic tree

The amino acid sequences of Crf4-3 homologs and related proteins were downloaded from the FungiDB (http://fungidb.org/fungidb) and InterPro (https://www.ebi.ac.uk/interpro/) websites. The DNA binding domain was obtained based on SMART-predicted analysis (http://smart.embl-heidelberg.de). Multiple sequence alignment was performed using a complete alignment method via DNAMAN software with default parameters. The protein pairwise similarity matrix was completed with parameters built-in via TBtools v2.106 (79). PhyloSuite 2 was used to conduct, manage, and streamline the phylogenic analyses with the help of several plug-in programs (80). iTOL was used for tree visualization and modification (81).

### Deletion of *crfA* in *A. fumigatus*

The *A. fumigatus* Afu8g04570 (*crfA*) mutation was generated by gene replacement via double homologous recombination events using the orotidine-5’-phosphate decarboxylase gene (*pyrG*) as a selectable marker (82). Briefly, the promoter (1616 bp) of *crfA*, the *pyrG* gene (3881 bp), the terminator (1599 bp) of *crfA*, and the plasmid backbone were amplified, respectively, using the primers *crfA*ko-up-F/R, *crfA*ko-pyrG-F/R, *crfA*ko-down-F/R, and pCSN43-kpni-hind-F/R. The obtained fragments were then assembled using a one-step cloning kit (ClonExpress Ultra One Step Cloning Kit; Vazyme, China), resulting in the plasmid pCSN-KO-*crfA*. The knockout fragment, including the promoter of *crfA*, the *pyrG* coding sequence, and the terminator of *crfA*, was amplified using the primers T7/3 and purified through gel extraction and then introduced into the *A. fumigatus* uracil-auxotrophic strain (CEA17) via protoplast transformation. Positive transformants were selected based on auxotrophic phenotype and verified by PCR. All primers used for construction and validation are listed in Table S2.

## Data Availability

The raw sequencing data for RNA-seq have been submitted to the Gene Expression Omnibus (GEO) with accession number GSE272185. All other relevant data are within the paper and its supplemental files.

## AFTER EPUB

*[This article was published on 13 December 2024 with errors in Fig. 4. The figure was corrected in the current version, posted on 23 December 2024.]*

## SUPPLEMENTAL MATERIAL

Data set S1 msphere.00703-24-s0001.xlsx FIMO analysis.

Data set S2 msphere.00703-24-s0002.xlsx RNA-seq data of all genes in WT and crf4-3KO strains with or without ketoconazole treatment.

Data set S3 msphere.00703-24-s0003.xlsx GSEA according to RNA-seq.

Supplemental material msphere.00703-24-s0004.docx Supplemental figures and tables.

ASM does not own the copyrights to Supplemental Material that may be linked to, or accessed through, an article. The authors have granted ASM a non-exclusive, world-wide license to publish the Supplemental Material files. Please contact the corresponding author directly for reuse.

## References

[1] Fisher MC, Gow NAR, Gurr SJ. 2016. Tackling emerging fungal threats to animal health, food security and ecosystem resilience. Philos Trans R Soc Lond B Biol Sci 371:20160332. doi:10.1098/rstb.2016.0332 28080997 PMC5095550

[2] Fisher MC, Gurr SJ, Cuomo CA, Blehert DS, Jin H, Stukenbrock EH, Stajich JE, Kahmann R, Boone C, Denning DW, Gow NAR, Klein BS, Kronstad JW, Sheppard DC, Taylor JW, Wright GD, Heitman J, Casadevall A, Cowen LE. 2020. Threats posed by the fungal kingdom to humans, wildlife, and agriculture. MBio 11:e00449-20. doi:10.1128/mBio.00449-20 32371596 PMC7403777

[3] Fisher M.C, Hawkins NJ, Sanglard D, Gurr SJ. 2018. Worldwide emergence of resistance to antifungal drugs challenges human health and food security. Science 360:739–742. doi:10.1126/science.aap7999 29773744

[4] Bongomin F, Gago S, Oladele RO, Denning DW. 2017. Global and multi-national prevalence of fungal diseases-estimate precision. J Fungi (Basel) 3:57. doi:10.3390/jof3040057 29371573 PMC5753159

[5] Stop neglecting fungi. 2017. Nat Microbiol 2:17120. doi:10.1038/nmicrobiol.2017.120 28741610

[6] Hosseini K, Ahangari H, Chapeland-Leclerc F, Ruprich-Robert G, Tarhriz V, Dilmaghani A. 2022. Role of fungal infections in carcinogenesis and cancer development: a literature review. Adv Pharm Bull 12:747–756. doi:10.34172/apb.2022.076 36415634 PMC9675916

[7] Narunsky-Haziza L, Sepich-Poore GD, Livyatan I, Asraf O, Martino C, Nejman D, Gavert N, Stajich JE, Amit G, González A, et al.. 2022. Pan-cancer analyses reveal cancer-type-specific fungal ecologies and bacteriome interactions. Cell 185:3789–3806. doi:10.1016/j.cell.2022.09.005 36179670 PMC9567272

[8] Saftien A, Puschhof J, Elinav E. 2023. Fungi and cancer. Gut 72:1410–1425. doi:10.1136/gutjnl-2022-327952 37147013

[9] Fisher MC, Henk D, Briggs CJ, Brownstein JS, Madoff LC, McCraw SL, Gurr SJ. 2012. Emerging fungal threats to animal, plant and ecosystem health. Nat New Biol 484:186–194. doi:10.1038/nature10947 PMC3821985 22498624

[10] Ekwomadu TI, Mwanza M. 2023. Fusarium fungi pathogens, identification, adverse effects, disease management, and global food security: a review of the latest research. Agr 13:1810. doi:10.3390/agriculture13091810

[11] Song J, Zhang S, Lu L. 2018. Fungal cytochrome P450 protein Cyp51: what we can learn from its evolution, regulons and Cyp51-based azole resistance. Fungal Biol Rev 32:131–142. doi:10.1016/j.fbr.2018.05.001

[12] Zhang J, Li L, Lv Q, Yan L, Wang Y, Jiang Y. 2019. The fungal CYP51s: their functions, structures, related drug resistance, and inhibitors. Front Microbiol 10:691. doi:10.3389/fmicb.2019.00691 31068906 PMC6491756

[13] Moye-Rowley WS. 2020. Linkage between genes involved in azole resistance and ergosterol biosynthesis. PLoS Pathog 16:e1008819. doi:10.1371/journal.ppat.1008819 32881974 PMC7470339

[14] Ferreira M da S, Malavazi I, Savoldi M, Brakhage AA, Goldman MHS, Kim HS, Nierman WC, Goldman GH. 2006. Transcriptome analysis of Aspergillus fumigatus exposed to voriconazole. Curr Genet 50:32–44. doi:10.1007/s00294-006-0073-2 16622700

[15] Liu X, Jiang J, Shao J, Yin Y, Ma Z. 2010. Gene transcription profiling of Fusarium graminearum treated with an azole fungicide tebuconazole. Appl Microbiol Biotechnol 85:1105–1114. doi:10.1007/s00253-009-2273-4 19820924

[16] da Silva Neto BR, Carvalho PFZ, Bailão AM, Martins WS, Soares CM de A, Pereira M. 2014. Transcriptional profile of Paracoccidioides spp. in response to itraconazole. BMC Genomics 15:254. doi:10.1186/1471-2164-15-254 24690401 PMC3975141

[17] Lupetti A, Danesi R, Campa M, Del Tacca M, Kelly S. 2002. Molecular basis of resistance to azole antifungals. Trends Mol Med 8:76–81. doi:10.1016/s1471-4914(02)02280-3 11815273

[18] Berman J, Krysan DJ. 2020. Drug resistance and tolerance in fungi. Nat Rev Microbiol 18:319–331. doi:10.1038/s41579-019-0322-2 32047294 PMC7231573

[19] Rogers PD. 2022. Sterol homeostasis in yeast. Nat Chem Biol 18:1170–1171. doi:10.1038/s41589-022-01143-y 36229682

[20] Maguire SL, Wang C, Holland LM, Brunel F, Neuvéglise C, Nicaud J-M, Zavrel M, White TC, Wolfe KH, Butler G. 2014. Zinc finger transcription factors displaced SREBP proteins as the major sterol regulators during Saccharomycotina evolution. PLoS Genet 10:e1004076. doi:10.1371/journal.pgen.1004076 24453983 PMC3894159

[21] Lee Y, Puumala E, Robbins N, Cowen LE. 2021. Antifungal drug resistance: molecular mechanisms in Candida albicans and beyond. Chem Rev 121:3390–3411. doi:10.1021/acs.chemrev.0c00199 32441527 PMC8519031

[22] Flowers SA, Barker KS, Berkow EL, Toner G, Chadwick SG, Gygax SE, Morschhäuser J, Rogers PD. 2012. Gain-of-function mutations in UPC2 are a frequent cause of ERG11 upregulation in azole-resistant clinical isolates of Candida albicans. Euk Cell 11:1289–1299. doi:10.1128/EC.00215-12 PMC3485914 22923048

[23] Hughes AL, Todd BL, Espenshade PJ. 2005. SREBP pathway responds to sterols and functions as an oxygen sensor in fission yeast. Cell 120:831–842. doi:10.1016/j.cell.2005.01.012 15797383

[24] Liu Z, Jian Y, Chen Y, Kistler HC, He P, Ma Z, Yin Y. 2019. A phosphorylated transcription factor regulates sterol biosynthesis in Fusarium graminearum. Nat Commun 10:1228. doi:10.1038/s41467-019-09145-6 30874562 PMC6420630

[25] Yin Y, Zhang H, Zhang Y, Hu C, Sun X, Liu W, et al.. 2021. Fungal Zn(II)_2_Cys_6_ transcription factor ADS-1 regulates drug efflux and ergosterol metabolism under antifungal azole stress. Antimicrob Agents Chemother 65:e01316-20. doi:10.1128/AAC.01316-20 33199382 PMC7848988

[26] Sun X, Wang K, Yu X, Liu J, Zhang H, Zhou F, Xie B, Li S. 2014. Transcription factor CCG-8 as a new regulator in the adaptation to antifungal azole stress. Antimicrob Agents Chemother 58:1434–1442. doi:10.1128/AAC.02244-13 24342650 PMC3957830

[27] Chen X, Xue W, Zhou J, Zhang Z, Wei S, Liu X, Sun X, Wang W, Li S. 2016. De-repression of CSP-1 activates adaptive responses to antifungal azoles. Sci Rep 6:19447. doi:10.1038/srep19447 26781458 PMC4726075

[28] Paul S, Verweij PE, Melchers WJG, Moye-Rowley WS. 2022. Differential functions of individual transcription factor binding sites in the tandem repeats found in clinically relevant cyp51A promoters in Aspergillus fumigatus. MBio 13:e0070222. doi:10.1128/mbio.00702-22 35467427 PMC9239056

[29] Du W, Zhai P, Wang T, Bromley MJ, Zhang Y, Lu L. 2021. The C_2_H_2_ transcription factor SltA contributes to azole resistance by coregulating the expression of the drug target Erg11A and the drug efflux pump Mdr1 in Aspergillus fumigatus. Antimicrob Agents Chemother 65:e01839-20. doi:10.1128/AAC.01839-20 33431412 PMC8097408

[30] Li Y, Dai M, Lu L, Zhang Y. 2023. The C_2_H_2_-type transcription factor ZfpA, coordinately with CrzA, affects azole susceptibility by regulating the multidrug transporter gene atrF in Aspergillus fumigatus. Microbiol Spectr 11:e00325-23. doi:10.1128/spectrum.00325-23 37318356 PMC10434176

[31] Zhao Y, Sun H, Li J, Ju C, Huang J. 2022. The transcription factor FgAtrR regulates asexual and sexual development, virulence, and DON production and contributes to intrinsic resistance to azole fungicides in Fusarium graminearum. Biol (Basel) 11:326. doi:10.3390/biology11020326 PMC8869466 35205191

[32] Moirangthem R, Kumar K, Kaur R. 2021. Two functionally redundant FK506-binding proteins regulate multidrug resistance gene expression and govern azole antifungal resistance. Antimicrob Agents Chemother 65:e02415-20. doi:10.1128/AAC.02415-20 33722894 PMC8316114

[33] Baker KM, Hoda S, Saha D, Gregor JB, Georgescu L, Serratore ND, Zhang Y, Cheng L, Lanman NA, Briggs SD. 2022. The Set1 histone H3K4 methyltransferase contributes to azole susceptibility in a species-specific manner by differentially altering the expression of drug efflux pumps and the ergosterol gene pathway. Antimicrob Agents Chemother 66:e0225021. doi:10.1128/aac.02250-21 35471041 PMC9112889

[34] Wurtele H, Tsao S, Lépine G, Mullick A, Tremblay J, Drogaris P, Lee E-H, Thibault P, Verreault A, Raymond M. 2010. Modulation of histone H3 lysine 56 acetylation as an antifungal therapeutic strategy. Nat Med 16:774–780. doi:10.1038/nm.2175 20601951 PMC4108442

[35] Henley MJ, Koehler AN. 2021. Advances in targeting 'undruggable' transcription factors with small molecules. Nat Rev Drug Discov 20:669–688. doi:10.1038/s41573-021-00199-0 34006959

[36] Yan C, Higgins PJ. 2013. Drugging the undruggable: transcription therapy for cancer. Biochim et Biophys Acta (BBA) - Rev on Cancer 1835:76–85. doi:10.1016/j.bbcan.2012.11.002 PMC3529832 23147197

[37] Chen A, Koehler AN. 2020. Transcription factor inhibition: lessons learned and emerging targets. Trends Mol Med 26:508–518. doi:10.1016/j.molmed.2020.01.004 32359481 PMC7198608

[38] Bushweller JH. 2019. Targeting transcription factors in cancer - from undruggable to reality. Nat Rev Cancer 19:611–624. doi:10.1038/s41568-019-0196-7 31511663 PMC8820243

[39] Tsafou K, Tiwari PB, Forman-Kay JD, Metallo SJ, Toretsky JA. 2018. Targeting Intrinsically disordered transcription factors: changing the paradigm. J Mol Biol 430:2321–2341. doi:10.1016/j.jmb.2018.04.008 29655986

[40] Huang Y, Li Y, Min J. 2024. Advances in inhibitor development targeting the PWWP domain. Trends Pharmacol Sci 45:193–196. doi:10.1016/j.tips.2024.01.007 38341359

[41] Vik A Rine J. 2001. Upc2p and Ecm22p, dual regulators of sterol biosynthesis in Saccharomyces cerevisiae. Mol Cell Biol 21:6395–6405. doi:10.1128/MCB.21.19.6395-6405.2001 11533229 PMC99787

[42] Stec I, Nagl SB, van Ommen GJ, den Dunnen JT. 2000. The PWWP domain: a potential protein-protein interaction domain in nuclear proteins influencing differentiation? FEBS Lett 473:1–5. doi:10.1016/s0014-5793(00)01449-6 10802047

[43] Rona GB, Eleutherio ECA, Pinheiro AS. 2016. PWWP domains and their modes of sensing DNA and histone methylated lysines. Biophys Rev 8:63–74. doi:10.1007/s12551-015-0190-6 28510146 PMC5425739

[44] Slater LM, Allen MD, Bycroft M. 2003. Structural variation in PWWP domains. J Mol Biol 330:571–576. doi:10.1016/s0022-2836(03)00470-4 12842472

[45] Wiles ET, Mumford CC, McNaught KJ, Tanizawa H, Selker EU. 2022. The ACF chromatin-remodeling complex is essential for Polycomb repression. Elife 11:e77595. doi:10.7554/eLife.77595 35257662 PMC9038196

[46] Kamei M, Ameri AJ, Ferraro AR, Bar-Peled Y, Zhao F, Ethridge CL, Lail K, Amirebrahimi M, Lipzen A, Ng V, Grigoriev IV, Schmitz RJ, Liu Y, Lewis ZA. 2021. IMITATION SWITCH is required for normal chromatin structure and gene repression in PRC2 target domains. Proc Natl Acad Sci U S A 118:e2010003118. doi:10.1073/pnas.2010003118 33468665 PMC7848606

[47] Qin S, Min J. 2014. Structure and function of the nucleosome-binding PWWP domain. Trends Biochem Sci 39:536–547. doi:10.1016/j.tibs.2014.09.001 25277115

[48] Wang Y, Reddy B, Thompson J, Wang H, Noma K, Yates JR 3rd, Jia S. 2009. Regulation of Set9-mediated H4K20 methylation by a PWWP domain protein. Mol Cell 33:428–437. doi:10.1016/j.molcel.2009.02.002 19250904 PMC2673476

[49] Gilbert TM, McDaniel SL, Byrum SD, Cades JA, Dancy BCR, Wade H, Tackett AJ, Strahl BD, Taverna SD. 2014. A PWWP domain-containing protein targets the NuA3 acetyltransferase complex via histone H3 lysine 36 trimethylation to coordinate transcriptional elongation at coding regions. Mol Cell Proteomics 13:2883–2895. doi:10.1074/mcp.M114.038224 25104842 PMC4223479

[50] Zhou J-X, Liu Z-W, Li Y-Q, Li L, Wang B, Chen S, He X-J. 2018. Arabidopsis PWWP domain proteins mediate H3K27 trimethylation on FLC and regulate flowering time. J Integr Plant Biol 60:362–368. doi:10.1111/jipb.12630 29314758

[51] Furukawa T, van Rhijn N, Fraczek M, Gsaller F, Davies E, Carr P, Gago S, Fortune-Grant R, Rahman S, Gilsenan JM, Houlder E, Kowalski CH, Raj S, Paul S, Cook P, Parker JE, Kelly S, Cramer RA, Latgé J-P, Moye-Rowley S, Bignell E, Bowyer P, Bromley MJ. 2020. The negative cofactor 2 complex is a key regulator of drug resistance in Aspergillus fumigatus. Nat Commun 11:427. doi:10.1038/s41467-019-14191-1 31969561 PMC7194077

[52] Shrivastava M, Kouyoumdjian GS, Kirbizakis E, Ruiz D, Henry M, Vincent AT, Sellam A, Whiteway M. 2023. The Adr1 transcription factor directs regulation of the ergosterol pathway and azole resistance in Candida albicans. MBio 14:e01807-23. doi:10.1128/mbio.01807-23 37791798 PMC10653825

[53] Silver PM, Oliver BG, White TC. 2004. Role of Candida albicans transcription factor Upc2p in drug resistance and sterol metabolism. Eukaryot Cell 3:1391–1397. doi:10.1128/EC.3.6.1391-1397.2004 15590814 PMC539032

[54] Huen MSY, Huang J, Leung JWC, Sy S-H, Leung KM, Ching Y-P, Tsao SW, Chen J. 2010. Regulation of chromatin architecture by the PWWP domain-containing DNA damage-responsive factor EXPAND1/MUM1. Mol Cell 37:854–864. doi:10.1016/j.molcel.2009.12.040 20347427 PMC3695488

[55] Chen TP, Tsujimoto N, Li E. 2004. The PWWP domain of Dnmt3a and Dnmt3b is required for directing DNA methylation to the major satellite repeats at pericentric heterochromatin. Mol Cell Biol 24:9048–9058. doi:10.1128/MCB.24.20.9048-9058.2004 15456878 PMC517890

[56] Guan M, Xia P, Tian M, Chen D, Zhang X. 2020. Molecular fingerprints of conazoles via functional genomic profiling of Saccharomyces cerevisiae. Toxicol In Vitro 69:104998. doi:10.1016/j.tiv.2020.104998 32919014

[57] Vary JC Jr, Gangaraju VK, Qin J, Landel CC, Kooperberg C, Bartholomew B, Tsukiyama T. 2003. Yeast Isw1p forms two separable complexes in vivo. Mol Cell Biol 23:80–91. doi:10.1128/MCB.23.1.80-91.2003 12482963 PMC140669

[58] Meng Y, Ni Y, Li Z, Jiang T, Sun T, Li Y, Gao X, Li H, Suo C, Li C, Yang S, Lan T, Liao G, Liu T, Wang P, Ding C. 2024. Interplay between acetylation and ubiquitination of imitation switch chromatin remodeler Isw1 confers multidrug resistance in Cryptococcus neoformans. Elife 13:e85728. doi:10.7554/eLife.85728 38251723 PMC10834027

[59] Tscherner M, Zwolanek F, Jenull S, Sedlazeck FJ, Petryshyn A, Frohner IE, Mavrianos J, Chauhan N, von Haeseler A, Kuchler K. 2015. The Candida albicans histone acetyltransferase Hat1 regulates stress resistance and virulence via distinct chromatin assembly pathways. PLoS Pathog 11:e1005218. doi:10.1371/journal.ppat.1005218 26473952 PMC4608838

[60] Khemiri I, Tebbji F, Burgain A, Sellam A. 2024. Regulation of copper uptake by the SWI/SNF chromatin remodeling complex in Candida albicans affects susceptibility to antifungal and oxidative stresses under hypoxia. FEMS Yeast Res 24:foae018. doi:10.1093/femsyr/foae018 38760885 PMC11160329

[61] Sharma C, Kadosh D. 2023. Post-transcriptional control of antifungal resistance in human fungal pathogens. Crit Rev Microbiol 49:469–484. doi:10.1080/1040841X.2022.2080527 35634915 PMC9766424

[62] Gsaller F, Hortschansky P, Furukawa T, Carr PD, Rash B, Capilla J, Müller C, Bracher F, Bowyer P, Haas H, Brakhage AA, Bromley MJ. 2016. Sterol biosynthesis and azole tolerance is governed by the opposing actions of SrbA and the CCAAT binding complex. PLoS Pathog 12:e1005775. doi:10.1371/journal.ppat.1005775 27438727 PMC4954732

[63] Paul S, Stamnes M, Thomas GH, Liu H, Hagiwara D, Gomi K, Filler SG, Moye-Rowley WS. 2019. AtrR is an essential determinant of azole resistance in Aspergillus fumigatus. MBio 10:e02563-18. doi:10.1128/mBio.02563-18 30862750 PMC6414702

[64] Henry KW, Nickels JT, Edlind TD. 2000. Upregulation of ERG genes in Candida species by azoles and other sterol biosynthesis inhibitors. Antimicrob Agents Chemother 44:2693–2700. doi:10.1128/AAC.44.10.2693-2700.2000 10991846 PMC90137

[65] Smith SJ, Crowley JH, Parks LW. 1996. Transcriptional regulation by ergosterol in the yeast Saccharomyces cerevisiae. Mol Cell Biol 16:5427–5432. doi:10.1128/MCB.16.10.5427 8816455 PMC231542

[66] Yang H, Tong J, Lee CW, Ha S, Eom SH, Im YJ. 2015. Structural mechanism of ergosterol regulation by fungal sterol transcription factor Upc2. Nat Commun 6:6129. doi:10.1038/ncomms7129 25655993

[67] Diao Y, Zhao R, Deng X, Leng W, Peng J, Jin Q. 2009. Transcriptional profiles of Trichophyton rubrum in response to itraconazole. Med Mycol 47:237–247. doi:10.1080/13693780802227308 18663659

[68] Zhang W, Yu L, Yang J, Wang L, Peng J, Jin Q. 2009. Transcriptional profiles of response to terbinafine in Trichophyton rubrum. Appl Microbiol Biotechnol 82:1123–1130. doi:10.1007/s00253-009-1908-9 19234875

[69] Hu C, Zhou M, Wang W, Sun X, Yarden O, Li S. 2018. Abnormal ergosterol biosynthesis activates transcriptional responses to antifungal azoles. Front Microbiol 9:9. doi:10.3389/fmicb.2018.00009 29387050 PMC5776110

[70] Cove DJ. 1966. The induction and repression of nitrate reductase in the fungus Aspergillus nidulans. Biochim Biophys Acta (BBA) - Enzymol Biol Oxid 113:51–56. doi:10.1016/S0926-6593(66)80120-0 5940632

[71] Margolin BS, Freitag M, Selker EU. 1997. Improved plasmids for gene targeting at the his-3 locus of Neurospora crassa by electroporation. Fungal Genet Rep 44:34–36. doi:10.4148/1941-4765.1281

[72] Aramayo R, Metzenberg RL. 1996. Gene replacements at the his-3 locus of Neurospora crassa. Fungal Genet Rep 43:9–13. doi:10.4148/1941-4765.1299 8953266

[73] Grant CE, Bailey TL, Noble WS. 2011. FIMO: scanning for occurrences of a given motif. Bioinformatics 27:1017–1018. doi:10.1093/bioinformatics/btr064 21330290 PMC3065696

[74] He Q, Cha J, He Q, Lee H-C, Yang Y, Liu Y. 2006. CKI and CKII mediate the FREQUENCY-dependent phosphorylation of the WHITE COLLAR complex to close the Neurospora circadian negative feedback loop. Genes Dev 20:2552–2565. doi:10.1101/gad.1463506 16980584 PMC1578678

[75] Hu C, Zhou M, Cao X, Xue W, Zhang Z, Li S, Sun X. 2022. Coordinated regulation of membrane homeostasis and drug accumulation by novel kinase STK-17 in response to antifungal azole treatment. Microbiol Spectr 10:e0012722. doi:10.1128/spectrum.00127-22 35196787 PMC8865411

[76] Freitas FZ, Virgilio S, Cupertino FB, Kowbel DJ, Fioramonte M, Gozzo FC, Glass NL, Bertolini MC. 2016. The SEB-1 transcription factor binds to the STRE motif in Neurospora crassa and regulates a variety of cellular processes including the stress response and reserve carbohydrate metabolism. G3 (Bethesda) 6:1327–1343. doi:10.1534/g3.116.028506 26994287 PMC4856084

[77] Luo CH, Loros JJ, Dunlap JC. 1998. Nuclear localization is required for function of the essential clock protein FRQ. EMBO J 17:1228–1235. doi:10.1093/emboj/17.5.1228 9482720 PMC1170471

[78] Ferraro AR, Lewis ZA. 2018. ChIP-seq analysis in *Neurospora crassa*. Edited by R. P. DeVries and A. Tsang. Fungal genomics: methods and protocols, Second Edition. Methods in Molecular Biology 1775:241–250. doi:10.1007/978-1-4939-7804-5_19 PMC10911069 29876822

[79] Chen C, Wu Y, Li J, Wang X, Zeng Z, Xu J, Liu Y, Feng J, Chen H, He Y, Xia R. 2023. TBtools-II: A "one for all, all for one"bioinformatics platform for biological big-data mining. Mol Plant 16:1733–1742. doi:10.1016/j.molp.2023.09.010 37740491

[80] Zhang D, Gao F, Jakovlić I, Zou H, Zhang J, Li WX, Wang GT. 2020. PhyloSuite: an integrated and scalable desktop platform for streamlined molecular sequence data management and evolutionary phylogenetics studies. Mol Ecol Resour 20:348–355. doi:10.1111/1755-0998.13096 31599058

[81] Letunic I, Bork P. 2024. Interactive Tree of Life (iTOL) v6: recent updates to the phylogenetic tree display and annotation tool. Nucleic Acids Res 52:W78–W82. doi:10.1093/nar/gkae268 38613393 PMC11223838

[82] D’Enfert C, Diaquin M, Delit A, Wuscher N, Debeaupuis JP, Huerre M, Latge JP. 1996. Attenuated virulence of uridine-uracil auxotrophs of Aspergillus fumigatus. Infect Immun 64:4401–4405. doi:10.1128/iai.64.10.4401-4405.1996 8926121 PMC174389

