# Supplementary material for: PWWP domain-containing protein Crf4-3 specifically modulates fungal azole susceptibility by regulating sterol C-14 demethylase ERG11
Source: mSphere. 2024 Dec 13;10(1):e00703-24. doi: 10.1128/msphere.00703-24 (PMC11774033; doi:10.1128/msphere.00703-24)
Supplement: Supplemental material — Supplemental figures and tables. [file msphere.00703-24-s0004.docx]

Supplemental Material - Figures and Tables

**
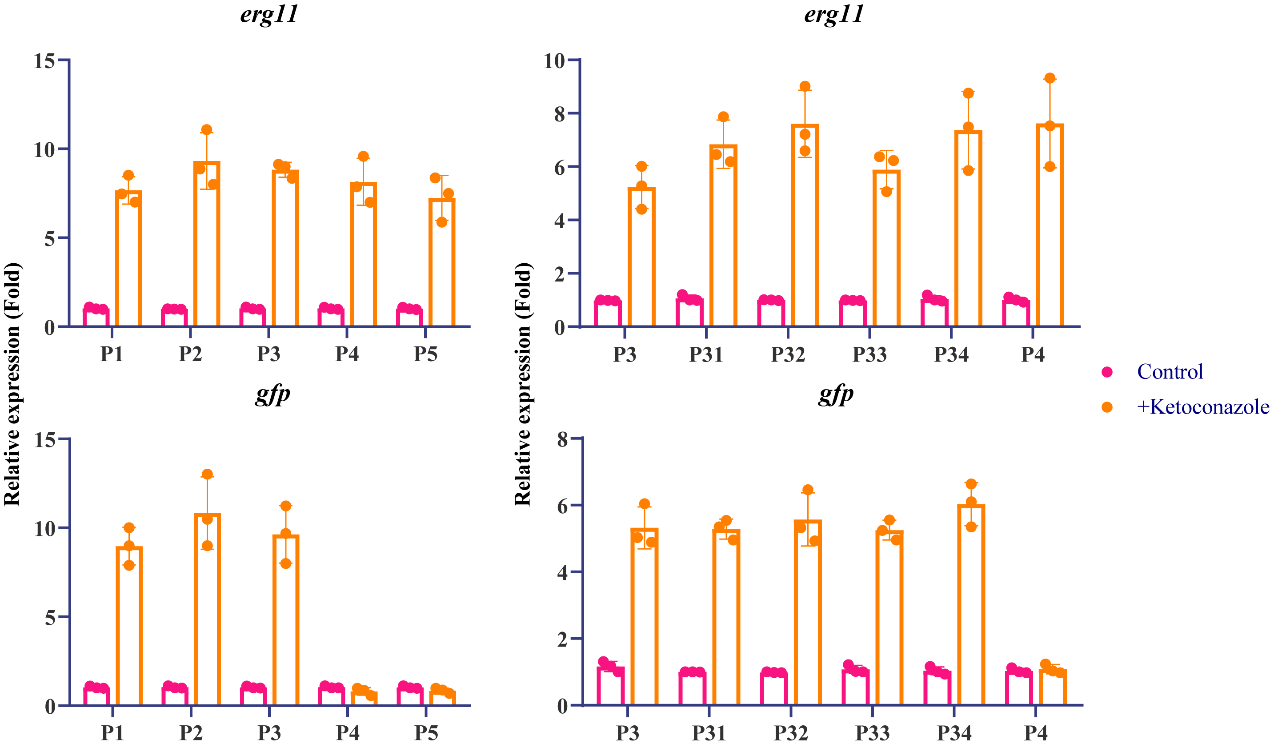
Supplemental Figure S1**

**Fig. S1 Identification of the key promoter region of *erg11* that is required for response to azoles in *N. crassa*.** (A) Gene expression of *erg11* and *gfp* in strains P1 (*gfp* driven by the -1328 to 0 region of the *erg11* promoter), P2 (*gfp* driven by the -1003 to 0 region of the *erg11* promoter), P3 (*gfp* driven by the -720 to 0 region of the *erg11* promoter), P4 (*gfp* driven by the -420 to 0 region of the *erg11* promoter), and P5 (*gfp* driven by the -268 to 0 region of the *erg11* promoter) after 12 hours of treatment with 1.5 μg/mL ketoconazole. (B) Gene expression detection of *erg11* and *gfp* in strains P3, P31 (*gfp* driven by the -669 to 0 region of the *erg11* promoter), P32 (*gfp* driven by the -598 to 0 region of the *erg11* promoter), P33 (*gfp* driven by the -541 to 0 region of the *erg11* promoter), P34 (*gfp* driven by the -468 to 0 region of the *erg11* promoter), and P4 after treatment with 1.5 μg/mL ketoconazole for 12 hours. Gene expression was detected using qRT-PCR. Expression levels were calculated using the 2^–ΔΔCT^ method with the expression of the *β-tubulin* gene as an internal reference. The expression level of each gene was normalized to 1 under untreated conditions. Error bars indicate standard deviations, n=3.


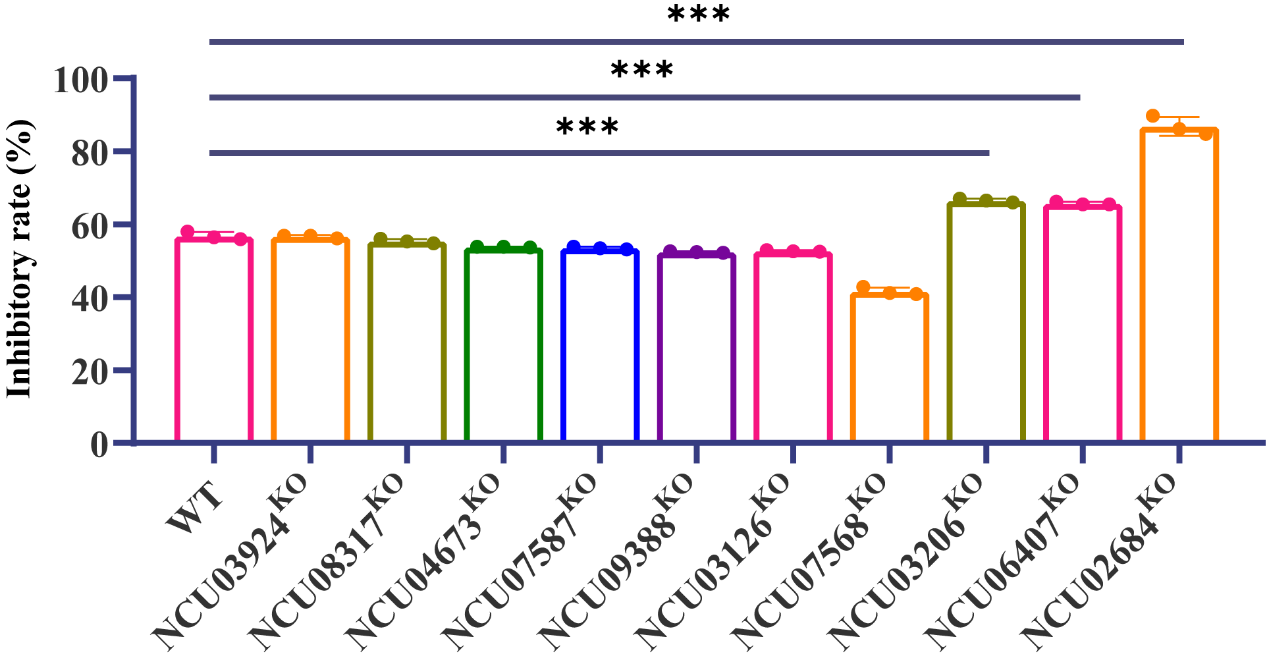
**Supplemental Figure S2**

**Fig. S2** **Rates of inhibition of the identified transcription factor mutants by ketoconazole (KTC).** The inhibition rate was calculated as 1 - [(colony diameter in drug plate/growth time) / (colony diameter in control plate/growth time)]. The levels of significance for differences between two strains are indicated by asterisks (*, P<0.05; **, P<0.01; **, P<0.001). *P*-values greater than 0.05 were considered not significant (ns) and were not annotated. Error bars indicate standard deviations, n=3.

**
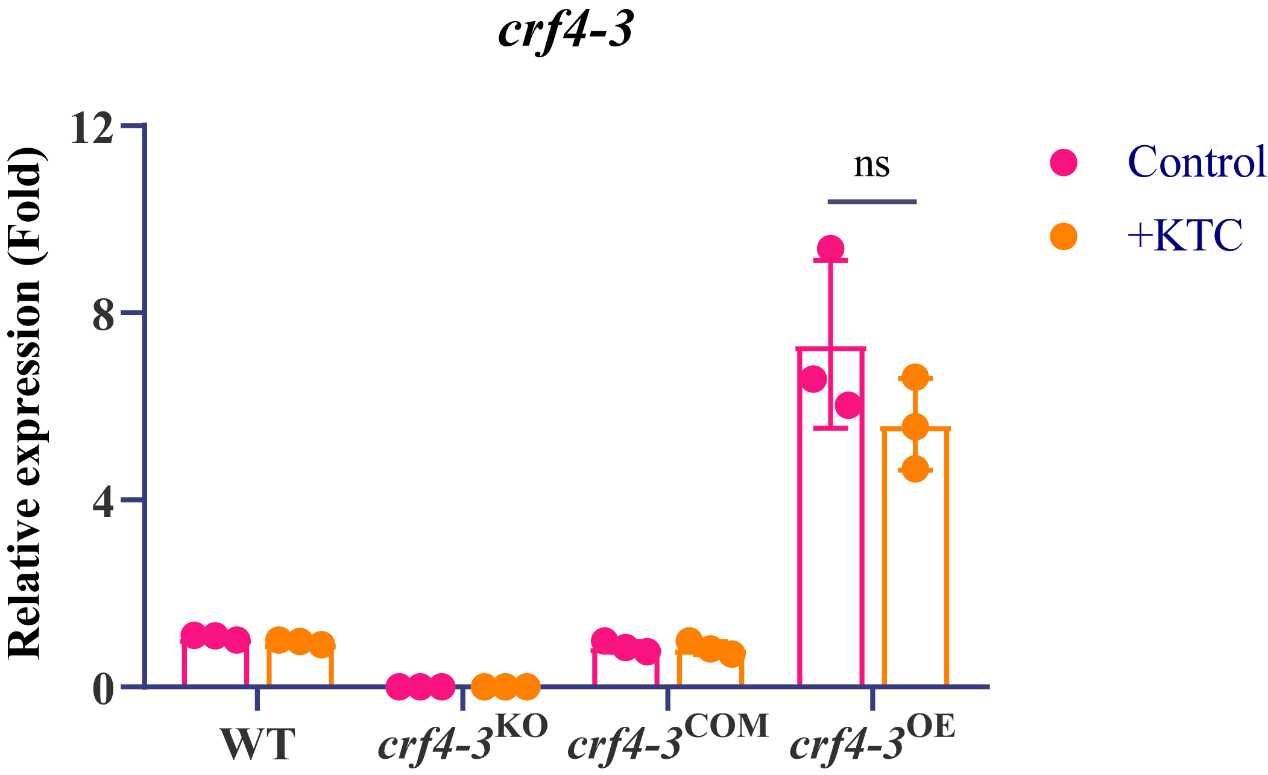
Supplemental Figure S3**

**Fig. S3** **Gene expression detection of *crf4-3* in *N. crassa crf4-3*^KO^, *crf4-3*^COM^, and *crf4-3*^OE^ strains treated with 1.5 μg/mL ketoconazole (KTC) for 12 hours.** Gene expression was detected using qRT-PCR. Expression levels were calculated using the 2^–ΔΔCT^ method with the expression of the *β-tubulin* gene as an internal reference. The expression level of *crf4-3* in the wild type without treatment was normalized to 1. The levels of significance for differences between two strains are indicated by asterisks (*, P<0.05; **, P<0.01; ***, P<0.001; ns, not significant, P>0.05). Error bars indicate standard deviations, n=3.

**
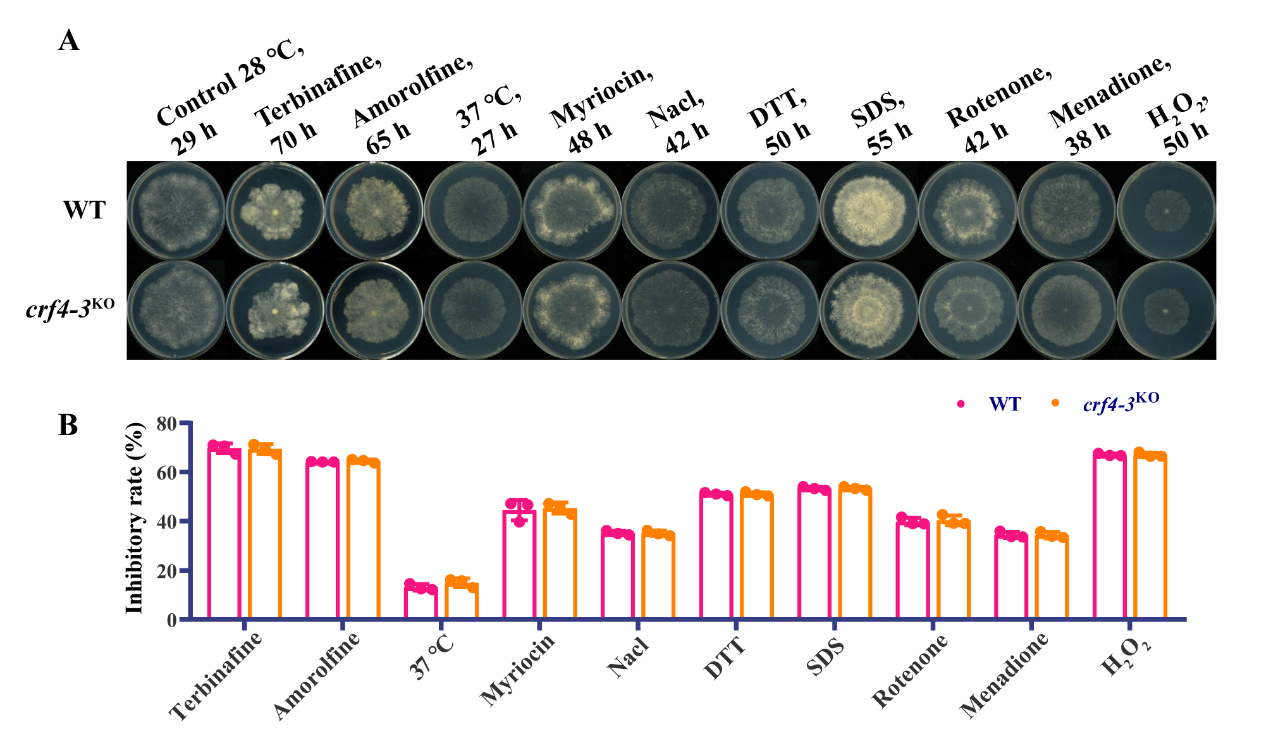
Supplemental Figure S4**

**Fig. S4 Sensitivity tests of WT and *crf4-3*^KO^ strains to other stresses.** (A) Sensitivity test of the wild type and *crf4-3*^KO^ strains to other stresses. A 2.5 μL conidial suspension (2×10^6^ spores/mL) of each strain was inoculated onto the center of Vogel’s plates with or without indicated compounds and incubated in the dark at 28°C. For heat stress, wild type and *crf4-3*^KO^ strain were inoculated onto the center of Vogel’s plates and incubated in the dark at 37°C. The concentrations of the compounds used were 2.5 µg/mL for terbinafine, 0.375 µg/mL for amorolfine, 20 ng/mL for Myriocin, 0.8 M for NaCl, 2 mM for DTT, 0.005% for SDS, 80 µg/mL for rotenone, 0.5 µg/mL for menadione, and 1.5 mM for H_2_O_2_. (B) Rates of inhibition of the wild type and *crf4-3*^KO^ strains by other stresses. The inhibition rate was calculated as 1 - [(colony diameter in drug plate/growth time) / (colony diameter in control plate/growth time)]. The levels of significance for differences between two strains are indicated by asterisks (*, P<0.05; **, P<0.01; **, P<0.001). *P*-values greater than 0.05 were considered not significant (ns) and were not annotated. Error bars indicate standard deviations, n=3.

**
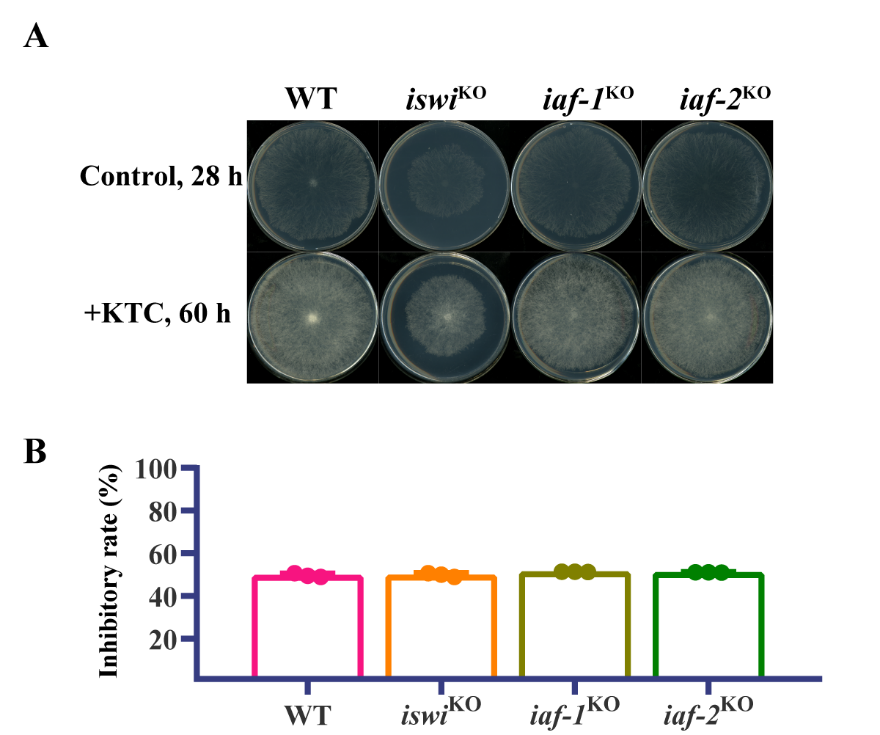
Supplemental Figure S5**

**Fig. S5 ISWI complex is not required for ketoconazole sensitivity.** (A) Drug sensitivity test of the wild type, *iswi*^KO^, *iaf-1*^KO^, and *iaf-2*^KO^ strains to ketoconazole. A 2.5 μL conidial suspension (2×10^6^ spores/mL) of each strain was inoculated onto the center of Vogel’s plates with or without ketoconazole (1.5 µg/mL) and incubated in the dark at 28°C. (B) Rates of inhibition of the wild type, *iswi*^KO^, *iaf-1*^KO^, and *iaf-2*^KO^ strains by ketoconazole. The inhibition rate was calculated as 1 - [(colony diameter in drug plate/growth time) / (colony diameter in control plate/growth time)]. The levels of significance for differences between two strains are indicated by asterisks (*, P<0.05; **, P<0.01; **, P<0.001). *P*-values greater than 0.05 were considered not significant (ns) and were not annotated. Error bars indicate standard deviations, n=3.

**
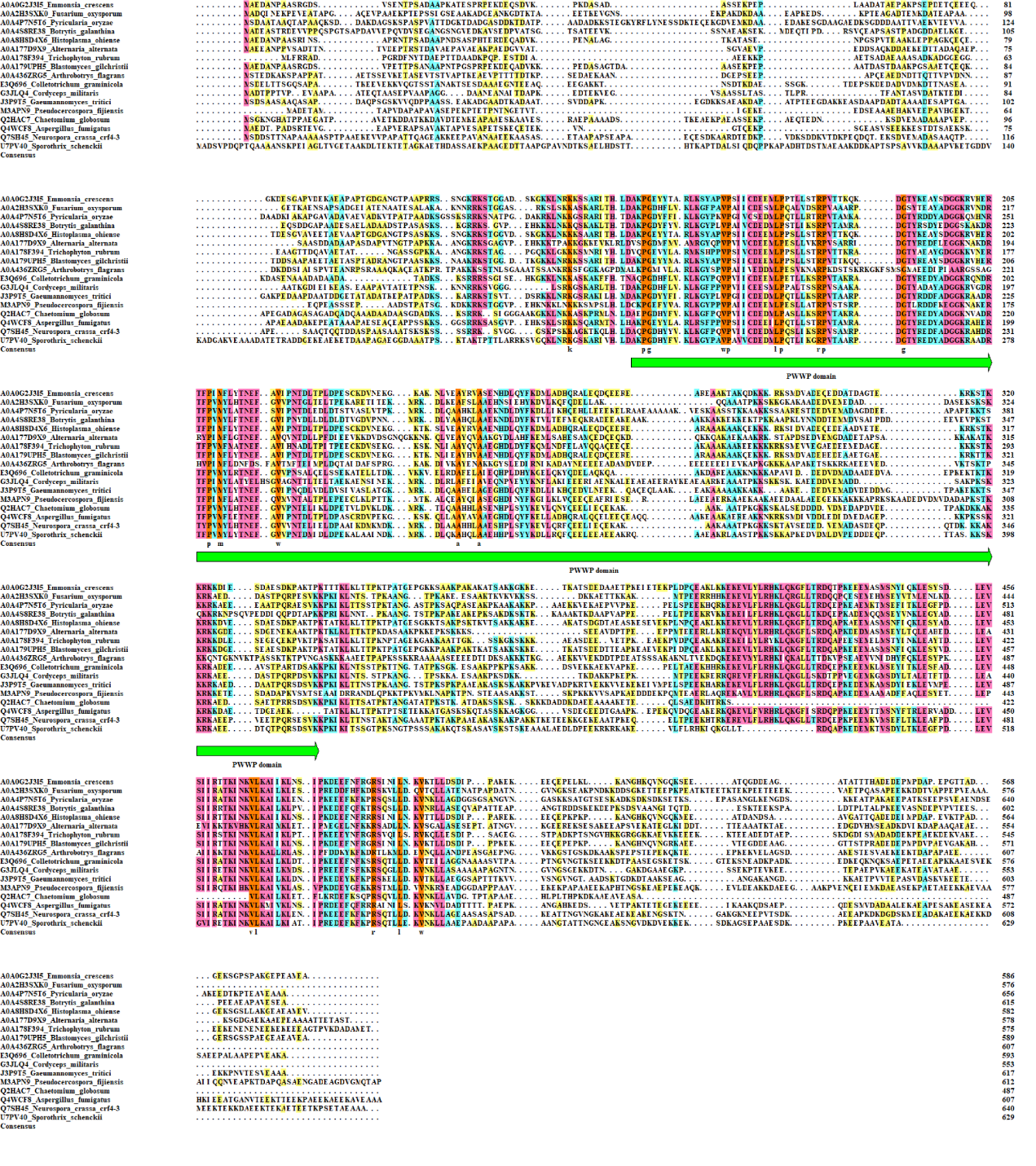
Supplemental Figure S6**

**Fig. S6 Multiple sequence alignment analysis of Crf4-3 homologs.** Multiple sequence alignment was performed using a complete alignment method via DNAMAN with default parameters. Protein similarity of 100% is indicated in orange, protein similarity greater than or equal to 75% is indicated in magenta, protein similarity greater than or equal to 50% is indicated in cyan, and protein similarity greater than or equal to 33% is indicated in yellow.

**Supplemental Table S1 A list of strains used in this study.**

| **Strains** | **Relevant Genotype** | **Source** | **Species** |
| --- | --- | --- | --- |
| WT | Wild type; *mat* a | FGSC#4200 | *Neurospora crassa* |
| WT | Wild type; *mat* A | FGSC#2225 |  |
| N85 | *bd Ku70^RIP^*; *mat* a | He *et al.*，2006 |  |
| *his-3*^–^ | *his-3^–^* | FGSC#6103 |  |
| P1 | P*erg11*(-1328 - 0)::*gfp*::T*trpC*::*his-3*^+^ | This study |  |
| P2 | P*erg11*(-1003 - 0)::*gfp*::T*trpC*::*his-3^+^* | This study |  |
| P3 | P*erg11*(-720 - 0)::*gfp*::T*trpC*::*his-3^+^* | This study |  |
| P4 | P*erg11*(-420 - 0)::*gfp*::T*trpC*::*his-3^+^* | This study |  |
| P5 | P*erg11*(-268 - 0)::*gfp*::T*trpC*::*his-3^+^* | This study |  |
| P31 | P*erg11*(-669 - 0)::*gfp*::T*trpC*::*his-3^+^* | This study |  |
| P32 | P*erg11*(-598 - 0)::*gfp*::T*trpC*::*his-3^+^* | This study |  |
| P33 | P*erg11*(-541 - 0)::*gfp*::T*trpC*::*his-3^+^* | This study |  |
| P34 | P*erg11*(-468 - 0)::*gfp*::T*trpC*::*his-3^+^* | This study |  |
| P341 | P*erg11*(-457 - 0)::*gfp*::T*trpC*::*his-3^+^* | This study |  |
| P342 | P*erg11*(-446 - 0)::*gfp*::T*trpC*::*his-3^+^* | This study |  |
| P343 | P*erg11*(-431 - 0)::*gfp*::T*trpC*::*his-3^+^* | This study |  |
| NCU02684^KO^(*crf4-3*^KO^) | Δ*NCU02684*::*Hph*^+^ | This study |  |
| *crf4-3*^COM^ | Δ*crf4-3*::*Hph*^+^::*crf4-3*::*Chl*^+^ | This study |  |
| sGFP*-crf4-3*^COM^ | Δ*crf4-3*::*Hph*^+^::*gfp*-*crf4-3*::*Chl*^+^ | This study |  |
| *crf4-3*^OE^ | Δ*crf4-3*::*Hph^+^*::P*tef-1*::*gfp*-*crf4-3*::T*trpC*::*Chl^+^* | This study |  |
| NCU03924^KO^ | Δ*NCU03924*::*Hph*^+^ | FGSC#21212 |  |
| NCU08317^KO^ | Δ*NCU08317*::*Hph*^+^; heterokaryon | FGSC#14853 |  |
| NCU04673^KO^ | Δ*NCU04673*::*Hph*^+^ | FGSC#17544 |  |
| NCU07587^KO^ | Δ*NCU07587*::*Hph*^+^ | FGSC#19413 |  |
| NCU09388^KO^(*iaf-2*^KO^) | Δ*NCU09388*::*Hph*^+^ | FGSC#18035 |  |
| NCU03126^KO^ | Δ*NCU03126*::*Hph*^+^; heterokaryon | FGSC#15480 |  |
| NCU07568^KO^ | Δ*NCU07568*::*Hph*^+^; heterokaryon | FGSC#12813 |  |
| NCU03206^KO^ | Δ*NCU03206*::*Hph*^+^; heterokaryon | FGSC#11486 |  |
| NCU06407^KO^ | Δ*NCU06407*::*Hph*^+^ | FGSC#11017 |  |
| *iswi*^KO^ | Δ*NCU03875*::*Hph*^+^ | FGSC#11780 |  |
| *iaf-1*^KO^ | Δ*NCU00412*::*Hph*^+^ | FGSC#12715 |  |
| CEA17 | *pyrG^-^* | Jin' Lab | *Aspergillus fumigatus* |
| CEA17^COM^ | *pyrG^+^* | Jin' Lab |  |
| *crfA*^KO^ | Δ*AFUA_8G04570*::*pyrG^+^* | This study |  |

**Supplemental Table S2 A list of PCR primers used in this study and their relevant characteristics.**

| **Sequences (5'--3')** | **Primer** | **Relevant Characteristics** |
| --- | --- | --- |
| GATAAGCTTGATATCGAATTCCTGTTTTCTTGCATGTCTGAACCAAG | P1-F | The construction of *gfp* reporter system in *N.crassa* |
| GATAAGCTTGATATCGAATTCAGTGGTGGTCAGCAAAGGCG | P2-F |  |
| GATAAGCTTGATATCGAATTCTTCATCGCTGCCCTGCATCTG | P3-F |  |
| GATAAGCTTGATATCGAATTCATGCGTTGCCCCTGCTCTT | P4-F |  |
| GATAAGCTTGATATCGAATTCCCACTGCACTGCCCTTGG | P5-F |  |
| GATAAGCTTGATATCGAATTCTCGCCGCGTTCTGAAGACG | P31-F |  |
| GATAAGCTTGATATCGAATTCTGCAAGACCAAAACAGCATGGC | P32-F |  |
| GATAAGCTTGATATCGAATTCCCGTCAGATGCGATTTCCCCC | P33-F |  |
| GATAAGCTTGATATCGAATTCGATGATCTGGAGAGGGTACGTACC | P34-F |  |
| GATAAGCTTGATATCGAATTCGAGGGTACGTACCGTCCAACC | P341-F |  |
| GATAAGCTTGATATCGAATTCCGTCCAACCAAAAATCCGAATACG | P342-F |  |
| GATAAGCTTGATATCGAATTCTCCGAATACGAATGCGTTGCC | P343-F |  |
| GCCCTTGCTCACCATGGTTGGAGAAGGAGGCGG | P*erg11*-R |  |
| ATGGTGAGCAAGGGCGAGGAG | sGFP-F |  |
| CGGCTCGAGGATATCGAATTCTTACTTGTACAGCTCGTCCATGC | sGFP-R |  |
| GAATTCGATATCCTCGAGCCGGGATCCACTTAACGTTACTGAA | TtrpC-F |  |
| TCCCCCGGGCTGCAGGAATTC GGCGTAGAGGATCCTCTAGAAAGAAG | TtrpC-R |  |
| CCGTCCAACCAAAAATCCGAATACGAATGCGTTGCCCCTGC | Probe-F | Probe used for the DNA pulldown |
| GCAGGGGCAACGCATTCGTATTCGGATTTTTGGTTGGACGG | Probe-R |  |
| GGCGAATTGGGTACCGCAAGACATCACTAGAGTTCC | *crf4-3*ko-up-F | Constructing plasmid pCSN-KO-*crf4-3* |
| ATATCATCTTCTGTCTGTGATGGAGTGGGTGAT | *crf4-3*ko-up-R |  |
| GACAGAAGATGATATTGAAGGAG | *crf4-3*ko-*hph*-F |  |
| CCCAAGATGTACAGGGATTTCAGTAACGTTAAGTGGAT | *crf4-3*ko-*hph*-R |  |
| CCTGTACATCTTGGGTTGAG | *crf4-3*ko-down-F |  |
| TTCGATATCAAGCTTTCGCTGATCTTCGTGTTTC | *crf4-3*ko-down-R |  |
| AAGCTTGATATCGAATTCCTGC | pCSN43-kpni-hind-F |  |
| GGTACCCAATTCGCCCTATAGT | pCSN43-kpni-hind-R |  |
| GGTTGTGGATGGATGGAAAG | *crf4-3*ko-up-verify-F | Validation of *N.crassa crf4-3*^KO^ transformants |
| CGATGCAAAGTGCCGATAA | *crf4-3*ko-up-verify-R |  |
| TGACGGCAATTTCGATGAT | *crf4-3*ko-down-verify-F |  |
| GACCAAGAAAGACCCTGATG | *crf4-3*ko-down-verify-R |  |
| CATTGCCAATCCCTCCATAC | *crf4-3*ko-het-verify-F |  |
| GGCGTTCTCTTCTTCCAATC | *crf4-3*ko-het-verify-R |  |
| GACGACAAGGTCACAGATAAG | gNCU02684F |  |
| GAGTTTCCCGTGCATCAA | gNCU02684R |  |
| GGCGAATTGGGTACCCGACTATACCATTCTCCACGATA | *crfA*ko-up-F | Constructing plasmid pCSN-KO-*crfA* |
| TTAGGAAGTCTAGAGAGTGTGCGACAGAGCTC | *crfA*ko-up-R |  |
| CTCTAGACTTCCTAATACCGCC | *crfA*ko-pyrG-F |  |
| CCGGATCTCTAGAGGTAAGTAATC | *crfA*ko-pyrG-R |  |
| CCTCTAGAGATCCGGACATAGGCACATATGGTTGTC | *crfA*ko-down-F |  |
| TTCGATATCAAGCTTACCTGCTTCAGTCAGGATA | *crfA*ko-down-R |  |
| TGGGACGATGACGAAGTA | *crfA*ko-up-verify-F | Validation of *A. fumigatus crfA*^KO^ transformants |
| ACAGAGCTGCAGGATAGT | *crfA*ko-up-verify-R |  |
| ACCTTGTCCTACGCTAGTAT | *crfA*ko-down-verify-F |  |
| CACGCTCTGTGCCTTTAT | *crfA*ko-down-verify-R |  |
| TCAAGAAGCTCCTCCATCT | gAf*crfA*-F |  |
| CTCTTTCTCTCCTTCGGTTTC | gAf*crfA*-R |  |
| CGCGGTGGCGGCCGCTCTAGTGCCAATCCCTCCATACA | *crf4-3*com-F | Constructing plasmid pCOM-*crf4-3* |
| TATAGGGCGAATTGGGTACCGGAACGCGTCTCAAACAA | *crf4-3*com-R |  |
| CGCGGTGGCGGCCGCTCTAGAGGTTGTGGATGGATGGAAAG | *crf4-3*com-P-F | Constructing plasmid pCOM-GFP-*crf4-3* |
| GCCCTTGCTCACCATGGTATGTGATGGAGTGGGT | *crf4-3*com-P-R |  |
| CATGGTGAGCAAGGGCGA | *crf4-3*com-sGFP-F |  |
| CGCCTCCGCCTCCGCCTCCCTTGTACAGCTCGTCCATGC | *crf4-3*com-sGFP-R |  |
| GCGGAGGCGGAGGCGGAGGCATGTCTGACGACTCCACGA | *crf4-3*com-*crf4-3*-T-F |  |
| CTATAGGGCGAATTGGGTACCGTACGGCTTCCAGGTAGTTA | *crf4-3*com-*crf4-3*-T-R |  |
| CGCGGTGGCGGCCGCTCTAGAACATATCCACCGTGACCACTGAACT | *crf4-3*oe-Ptef-1-F | Constructing plasmid pOE-GFP-*crf4-3* |
| CGCGCCTTTGACGGTTGATGTGCTGACTGG | *crf4-3*oe-Ptef-1-R |  |
| ACATCAACCGTCAAAGGCGCGATGGTGAGCAAGGGCGAGGAG | *crf4-3*oe-sGFP-F |  |
| CGCCTCCGCCTCCGCCTCCCTTGTACAGCTCGTCCATGCCG | *crf4-3*oe-sGFP-R |  |
| GCGGAGGCGGAGGCGGAGGCATGTCTGACGACTCCACGA | *crf4-3*oe-*crf4-3*-F |  |
| GATCCCGGCTCGAGGATATCTCAGGCAGCAGCCTCAG | *crf4-3*oe-*crf4-3*-R |  |
| GATATCCTCGAGCCGGGATCCACTTAACGTTACTGAA | *crf4-3*oe-TtrpC-F |  |
| CTATAGGGCGAATTGGGTACCGGCGTAGAGGATCCTCTAGAAAGAAG | *crf4-3*oe-TtrpC-R |  |
| GTTTGGATGATCTGGAGAGG | P1-F | The *erg11* promoter regions of *N.crassa* were ampliﬁed with the primers for ChIP-qPCR analysis |
| GTCTCCCAGCCAATCATAAG | P1-R |  |
| CCTCCTCGGAAAGAAGACTA | P2-F |  |
| TAAATCTCTTCGGCGTTGAC | P2-R |  |
| CCATCGAGCACGGTGTTGTCA | qACT-AF04740F | Quantitative real-time PCR primers for analysis of *actin* expression in *A.fumigatus* |
| AAGGCGGGAGCGTTGAAGGT | qACT-AF04740R |  |
| CCGTCCATGCTCCTGACCTTGA | q6-AF03630F | Quantitative real-time PCR primers for analysis of *erg6* expression *in A.fumigatus* |
| ACCATCACCCAGCTCGATACCC | q6-AF03630R |  |
| TCGGATCGGACGTGGTGTATGA | q11A-AF06890F | Quantitative real-time PCR primers for analysis of *erg11A* expression *in A.fumigatus* |
| CAGCCATTGCCGCAGAGATGT | q11A-AF06890R |  |
| TGCCAGGTCTGAGGAGCACTTC | q11B-AF03740F | Quantitative real-time PCR primers for analysis of *erg11B* expression *in A.fumigatus* |
| CCAATGCACCTATGCCGTCCAG | q11B-AF03740R |  |
| CCCAAGAACATGATGGCTGCTTCT | qTUB-NCU04054F | Quantitative real-time PCR primers for analysis of *tubulin* expression in *N.crassa* |
| TTGTTCTGAACGTTGCGCATCTGG | qTUB-NCU04054R |  |
| AAATCGATTACGGCTACGGTCTCG | q11-NCU02624F | Quantitative real-time PCR primers for analysis of *erg11* expression in *N.crassa* |
| TATCGCTACCATCCACGTTCCTGA | q11-NCU02624R |  |
